# Membrane associated cancer-oocyte neoantigen SAS1B/ovastacin is a candidate immunotherapeutic target for uterine tumors

**DOI:** 10.18632/oncotarget.4734

**Published:** 2015-08-18

**Authors:** Eusebio S. Pires, Ryan S. D'Souza, Marisa A. Needham, Austin K. Herr, Amir A. Jazaeri, Hui Li, Mark H. Stoler, Kiley L. Anderson-Knapp, Theodore Thomas, Arabinda Mandal, Alain Gougeon, Charles J. Flickinger, David E. Bruns, Brian A. Pollok, John C. Herr

**Affiliations:** ^1^ Department of Cell Biology at the School of Medicine, University of Virginia, Charlottesville, Virginia, USA; ^2^ Department of Pathology at the School of Medicine, University of Virginia, Charlottesville, Virginia, USA; ^3^ Department of Obstetrics and Gynecology at the School of Medicine, University of Virginia, Charlottesville, Virginia, USA; ^4^ Neoantigenics, Inc, Charlottesville, Virginia, USA; ^5^ CRCL, UMR Inserm-1052, CNRS-5286, Faculté de Médecine Laënnec, Lyon, France

**Keywords:** ASTL/SAS1B/ovastacin, cancer surface neoantigen, oocyte-specific protein, uterine tumor biomarkers, immunotherapy target

## Abstract

The metalloproteinase SAS1B [ovastacin, ASTL, astacin-like] was immunolocalized on the oolemma of ovulated human oocytes and in normal ovaries within the pool of growing oocytes where SAS1B protein was restricted to follicular stages spanning the primary-secondary follicle transition through ovulation. Gene-specific PCR and immunohistochemical studies revealed ASTL messages and SAS1B protein in both endometrioid [74%] and malignant mixed Mullerian tumors (MMMT) [87%] of the uterus. A MMMT-derived cell line, SNU539, expressed cell surface SAS1B that, after binding polyclonal antibodies, internalized into EEA1/LAMP1-positive early and late endosomes. Treatment of SNU539 cells with anti-SAS1B polyclonal antibodies caused growth arrest in the presence of active complement. A saporin-immunotoxin directed to SAS1B induced growth arrest and cell death. The oocyte restricted expression pattern of SAS1B among adult organs, cell-surface accessibility, internalization into the endocytic pathway, and tumor cell growth arrest induced by antibody-toxin conjugates suggest therapeutic approaches that would selectively target tumors while limiting adverse drug effects in healthy cells. The SAS1B metalloproteinase is proposed as a prototype cancer-oocyte tumor surface neoantigen for development of targeted immunotherapeutics with limited on-target/off tumor effects predicted to be restricted to the population of growing oocytes.

## INTRODUCTION

SAS1B (sperm acrosomal SLLP1 binding protein, a.k.a ovastacin, astacin-like or ASTL, GenBank ID NM_001002036.3), is a cortical granule and oocyte surface-associated zinc matrix metallo-proteinase (MMP, EC 3.4.24.21) with reported roles in sperm-egg interaction [1, 2] and in the block to polyspermy [3] during eutherian fertilization. Beginning at its N-terminus this ∼46 kDa metalloproteinase consists of a signal peptide, pro-peptide motif, proteinase domain containing an active hex-box [HEXXHXXGXXH) catalytic site motif, and a unique C terminus [2]. The pattern of SAS1B protein expression within the ovary is conserved in several mammalian groups including non-human primates, canines, felines, artiodactyls, and rodents where SAS1B protein is restricted to the pool of growing oocytes beginning within primary-secondary transition follicles and persisting through ovulation [2]. Other than the ovary, no normal adult tissues appear to express SAS1B: upon interrogation of the murine EST database [4], ASTL ESTs are annotated only in ovary, ova, zygote, and early embryo [2, 1]. Interrogation of the NCBI human EST database did reveal a single EST in human uterus [5], but detailed examination of this GENBANK deposit revealed that the cDNA sequence was from an un-classified human uterine tumor. Based on this information, the current study was undertaken to investigate the frequency of ASTL transcription and translation in an exploratory cohort of uterine tumors and to evaluate if SAS1B might offer opportunities as a target for a novel immunotherapeutic approach.

Uterine cancer is the most prevalent gynecologic malignancy and the 4^th^ most prevalent cancer among United States women, with 54,870 new cases and 10,170 deaths predicted in 2015, most occurring in women over 55 years of age [6]. The present study focused on malignant mixed Mullerian tumors (MMMT, aka carcinosarcomas) and on endometrioid cancers. Although the most common endometrial cancer cell type is endometrioid adenocarcinoma, which is composed of malignant glandular epithelial elements, uterine carcinosarcomas were found to have the higher incidence of SAS1B expression in this study. Carcinosarcomas, or MMMT, are very aggressive, rare, biphasic tumors composed of epithelial and mesenchymal elements believed to have a monoclonal origin. Uterine carcinosarcomas have an estimated recurrence rate of 40–60% [7], with approximately 35% of patients having extra-uterine disease at diagnosis. Patients may undergo comprehensive surgical excision of the tumor followed by adjuvant therapy in the forms of chemotherapy alone or chemo-radiotherapy in combination [8–11]. At present there are neither routine biomarkers used to diagnose nor a targeted immunotherapy for any uterine cancer. Results of two randomized trials on the use of external-beam radiation therapy (EBRT) in patients with stage I disease did not show improved survival but did show reduced locoregional recurrence (3%–4% vs. 12%–14% after 5–6 years’ median follow-up, *P* < 0.001), however with this radiation therapy an increase in adverse side effects was observed [12, 13, 14]. Vaginal cuff brachytherapy is associated with less radiation-related morbidity than is EBRT and has been shown to be equivalent to EBRT in the adjuvant setting for patients with stage I disease [15].

The advent of effective, rationally designed, targeted antibody-drug conjugates such as gentuzumab ozogamicin targeting CD33 for acute myeloid leukemia [16], trastuzumab-emtansine (TDM-1, Kadcyla) targeting Her2 for breast cancer [17], and brentuximab vedotin (Adcetris) targeting CD30 for Hodgkin's lymphoma and for systemic anaplastic large cell lymphoma [18] has stimulated a search for novel drug targets that provide new opportunities and paradigms for immunotherapeutic intervention [19]. In the following studies attributes of SAS1B are defined that support its candidacy as a tumor cell-specific target antigen, including tumor cell-surface accessibility, immunogenicity, internalization of immune complexes into the endosomal-lysosomal system, and immunotoxin delivery resulting in tumor cell growth arrest *in vitro*. SAS1B is expressed in endometrioid and MMMT uterine cancers at high incidence while lacking expression in normal tissues with the exception of growing oocytes. Taken together these features make SAS1B an attractive tumor surface target for immunotherapy.

## RESULTS

### SAS1B is an ovary-specific protein with expression restricted to oocytes in growing follicles

**Figure 1:**
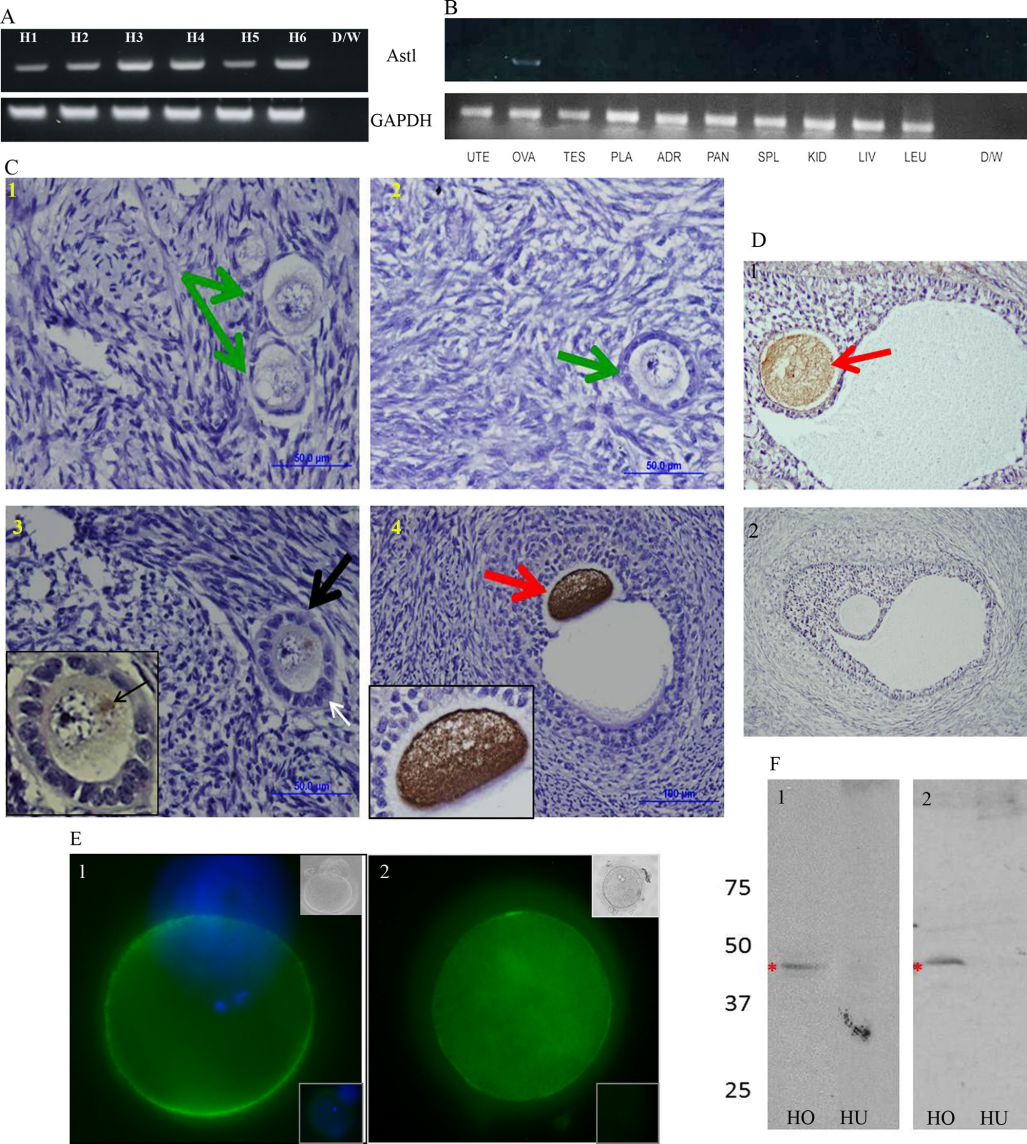
ASTL/SAS1B has ovary and oocyte restricted expression among normal human tissues, and a population of SAS1B is found on the oolemma of ovulated human oocytes **Panel A:** RT-PCR analyses of 6 normal human ovarian cDNAs (H1-H6) using a c-terminus ASTL specific primer set show 309 base pair ASTL amplicons in each specimen. **Panel B:** ASTL expression was restricted to ovarian specimens (lane OVA) while other tissues (uterus, testes, placenta, adrenal, pancreas, spleen, kidney, liver and leukocytes) were ASTL silent. GAPDH was used as a loading control for all RT-PCR assays. **Panels C and D:** Immunohistochemical localization of SAS1B in human ovarian tissue sections with anti-SAS1B antibodies (C- IM antibodies and D1- PPpAb, D2- PIM antibodies/ normal rabbit IgG). SAS1B protein was localized to oocytes in primary-secondary transition follicles (C3), early antral follicles (C4) and beyond (D1). Oocytes within primordial follicles (C1) and small primary follicles i.e. resting follicles (C2) did not express SAS1B (green arrows). An early developing primary-secondary transition follicle (black arrow, C3, with development of a bilaminar layer noted at white arrow) shows initiation of SAS1B expression in the perinuclear region of the oocyte (inset) presumably in the Golgi apparatus. **Panel E:** Live stained fertilized human oocytes show membrane staining of SAS1B by immunofluorescence. Top inset shows phase image and bottom inset shows PIM control. **Panel F:** Western blots reveal a 46 kDa SAS1B protein (red asterisk) in human ovarian protein extracts (HO) with IM antibodies (F1, HO) and PPpAb (F2, HO). In protein extracts from normal human uterus (F1, HU and F2, HU) SAS1B was not detected.

SAS1B has been shown to be restricted to growing oocytes in secondary follicles and subsequent stages through ovulation in ovaries of several eutherian orders [2]. In humans, where mono-ovular menstrual cycles occur, the relative abundance of SAS1B mRNAs isolated from whole ovaries is predicted to be low, since the pool of growing oocytes in secondary follicle stages and beyond is small and many secondary follicles undergo atresia [20]. When SAS1B expression in human ovaries was evaluated, 35 cycles of RT-PCR using a panel of ovarian cDNAs revealed ASTL amplicons in each of 6 different specimens (Figure 1A: H1-6). These amplicons were cloned and sequenced, and showed 99% identity to the ASTL reference sequence confirming SAS1B expression in normal ovary. RT-PCR of a panel of normal human tissues identified ASTL amplicons only in the ovary (Figure 1B).

Using both a rabbit polyclonal antibody generated to recombinant human SAS1B (IM) (Figure 1C, IM) and a commercial ASTL pro-peptide antibody (Figure 1D), SAS1B was immunolocalized in human ovarian sections within oocytes in a early primary to secondary transition follicle (Figure 1C3 and inset, black arrow, where perinuclear staining was first detected), secondary follicles (red arrows as in C4 and D1) and subsequent stages but not in the ovarian reserve of primordial follicles (green arrows, C1), in primary follicles (C2), or in any other ovarian cell type. Pre-immune (PIM) or normal rabbit IgG control antibodies did not stain the specimens (represented in panel D2). Immunofluorescent staining of two live fertilized human oocytes (Figure 1, panels Figure 1E1 and 1E2) revealed the presence of SAS1B^+^ domains (green stain) exposed on the oolemma. Western blot analysis using commercially available normal human ovarian (HO) and normal human uterine (HU) extracts probed with Rb IM (Figure 1F1) and Rb ASTL propeptide pAb, PPpAb (Figure 1F2) revealed immunoreactive bands at 46 kDa in normal human ovary but not in normal uterus with either of the antibodies. PIM showed no immunostaining to any proteins (Supplementary Figure S1, blot 1) and GAPDH was used to show equal loading (Supplementary Figure S1, blot S2, control).

### Endometrial tumors transcribe the ASTL gene and translate SAS1B proteins at high incidence while normal endometrium lacks SAS1B expression

**Figure 2:**
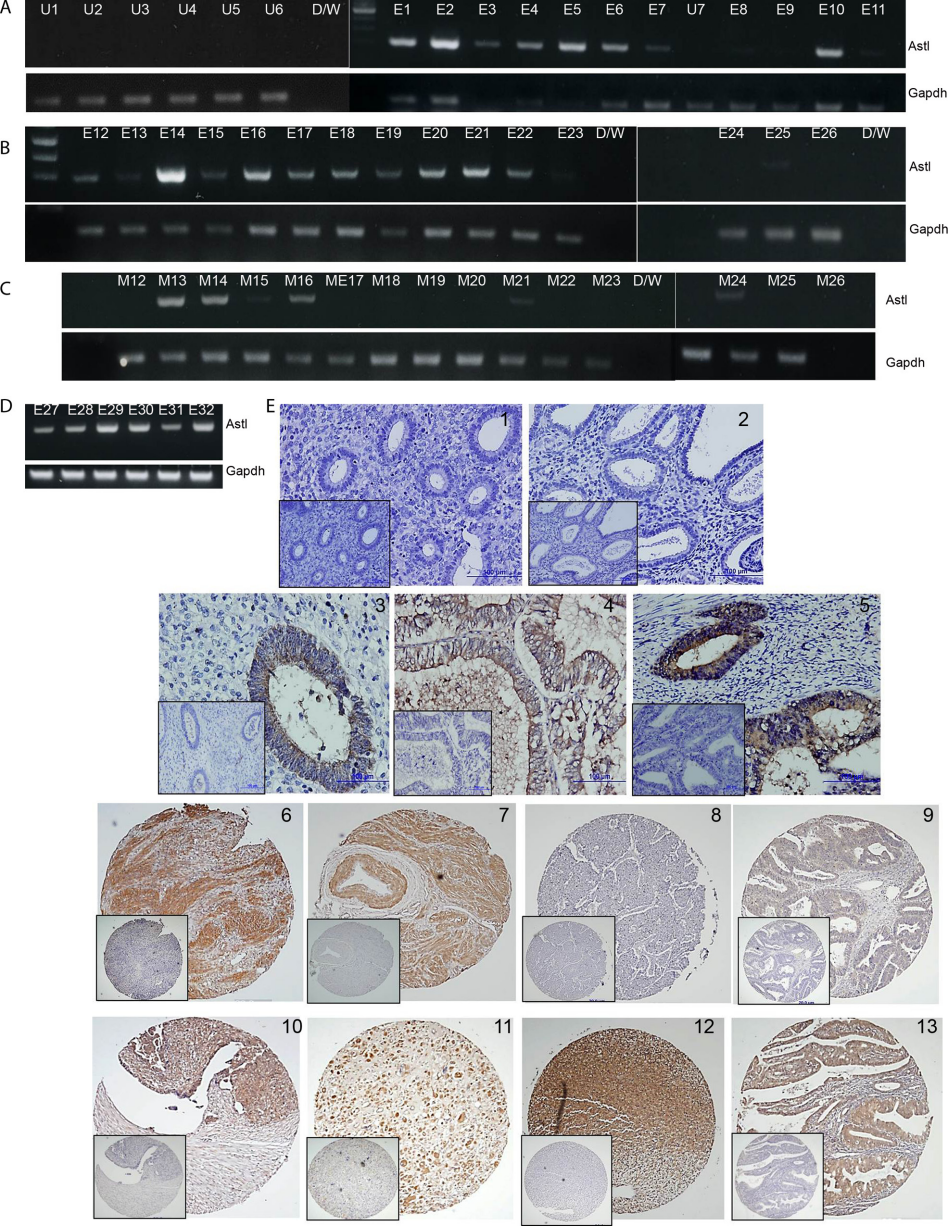
SAS1B expression occurs at high incidence in uterine tumors **Panel A:** Normal human uterine cDNA samples U1–U7 show no ASTL expression (lack of 309 base pair amplimer). Representative images indicating SAS1B expression in tumors; E1–7 (grade 1 endometrioid tumors) are ASTL positive, while in E8–11 (grade 3 endometrioid tumors) only E10 is positive. **Panel B:** In this group of 15 uterine tumors (E12–E26) 11 were positive for ASTL including 10 endometrioid specimens and one MMMT (E13). **Panel C:** cDNAs from myometrial tissue adjacent to the tumor (M12–26) show 5/15 positivity, likely due to tumor infiltration. **Panel D:** E27, 28, 30 and 32 are endometrioid tumors (4/4 positive) and E29 and 31 are MMMT (2/2 positive). **Panel E:** IHC using the IM or PIM (inset) antibodies on normal human proliferative (1) or secretory (2) endometrium showing absence of SAS1B protein. Immunohistochemical staining of SAS1B (brown DAB reaction product) in endometrioid cancers grade 1 (Panels 3–5), endometrioid cancers grade 3 (Panels 6–9), and MMMT's (Panels 10–13).

RNA samples from 38 uterine tumor specimens and 8 normal uterine biopsy tissues were converted to cDNAs and screened for SAS1B transcripts using C-term ASTL primers. None of the normal uterine biopsy specimens showed evidence of SAS1B transcripts (Figure 2A: U1–U7; Figure 1B: UTE) However, 30/38 (78.9%) uterine tumors tested positive for ASTL RNA (Figure 2, panels A–D) including specimens of grade 1 and grade 3 endometrioid cancers, serous papillary carcinoma, and MMMT. Amplicons were cloned and sequenced revealing 99% identify to the reference sequence with only an occasional SNP. Specificity of the primers in the PCR reaction was validated (Supplementary Figure S2). Paraffin embedded tumor samples were investigated by IHC for SAS1B protein in whole block sections and tissue microarrays (Figure 2E). Specimens of normal proliferative and secretory phase endometrium (Figure 2E1 and 2E2, respectively) did not stain for SAS1B protein by IHC in either glands or stroma. 14 of 21 endometrioid carcinomas (∼66%) tested positive by immunohistochemistry, with SAS1B protein localized to the compartment of the adenocarcinomatous glands (Figure 2E 3–9). In some samples (Figure 2E4) the apical surface of the epithelial sheet was prominently SAS1B positive and SAS1B immunoreactivity was noted in the glandular lumen. In the case of the MMMT specimens, 12 of 14 (∼85%) tested positive for SAS1B (Figure 2E 10–13). Cellular staining was not uniform, in some specimens the strongest staining was seen in the cytoplasm of mesenchymal cells (panels 11, 12), whereas other specimens displayed cytoplasmic staining of both epithelial and mesenchymal components (panels 10 and 13).

### ASTL transcription and translation in cell lines derived from uterine MMMT

**Figure 3:**
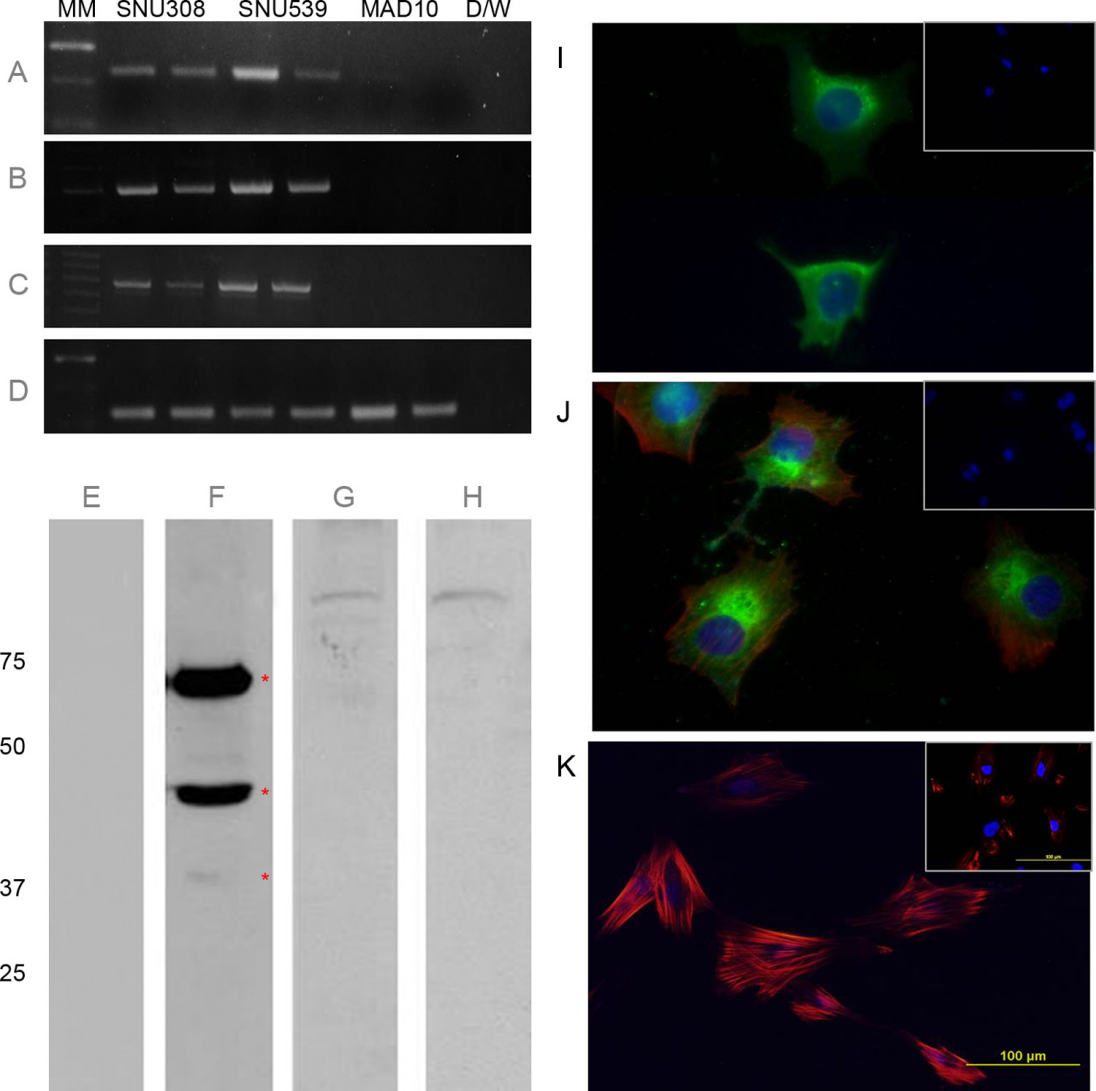
ASTL/SAS1B expression in MMMT derived cell lines cDNAs from MMMT derived cell lines S08-38710 and SNU539 along with control MAD10 cells were probed (in duplicate) for ASTL using domain specific primers (**Panels A, B, C** represent ASTL amplicons of 237 base pair N-terminus, 309 base pair C-terminus and 579 base pair catalytic region, respectively). **Panel D:** ASTL transcription was noted in both S08-38710 and SNU539 while MAD10 was ASTL negative. GAPDH was used as a loading control. **Panel F-H:** On Western blots, major immunoreactive SAS1B protein isoforms (red asterisks, F) of ∼46 kDa [predicted from primary sequence] and ∼65 kDa, and a less abundant ∼36-37 kDa band, were identified in Celis extracts of SNU539 cells probed with IM antibodies, while blots exposed to PIM antibodies (E) were negative. MAD10 protein extracts showed no specific immunoreactivity with IM antibodies (H) or control PIM antibodies (G). In fixed S08-38710 **(Panel I)** and SNU539 **(Panel J)** cells IM antibodies localized SAS1B in the cytoplasm with high concentrations noted in the perinuclear region (Green is SAS1B, blue is DAPI nuclear counterstain, red is phalloidin counterstain for actin). No SAS1B immunoreactivity was noted in MAD10 cells **(Panel K)**. Insets (I-K) show lack of immunoreactivity with PIM antibody on the respective cell types.

Since IHC indicated that the SAS1B protein was translated at high incidence in primary uterine MMMTs, cell lines (SNU539 and S08-38710) derived from this tumor type were employed for subsequent experiments, while MAD10-252/616 was tested for SAS1B as a potential negative control. cDNAs from these 3 lines were examined for ASTL messages using 3 primer sets designed against different domains of the ASTL transcript viz., N-terminus, C-terminus, and catalytic regions (Figure 3A, 3B and 3C respectively). ASTL amplicons were identified only in the MMMT cell lines and were cloned and confirmed to be ASTL with 99% identity by DNA sequencing. Western blot analysis revealed protein microheterogeneity of SAS1B species in the SNU539 lysate. The IM antibody identified a 46 kDa band, the mass deduced from the primary sequence, as well as ∼65 kDa and fainter ∼36–37 kDa immunoreactive bands (Figure 3F). These immunoreactive bands were not seen with PIM antibodies (Figure 3E). Protein extracts of MAD10-252/616 showed no SAS1B immunoreactivity (Panel G for IM and H for PIM).

### SAS1B protein is expressed within the cytoplasm and appears on the plasma membrane of uterine cancer cells

**Figure 4:**
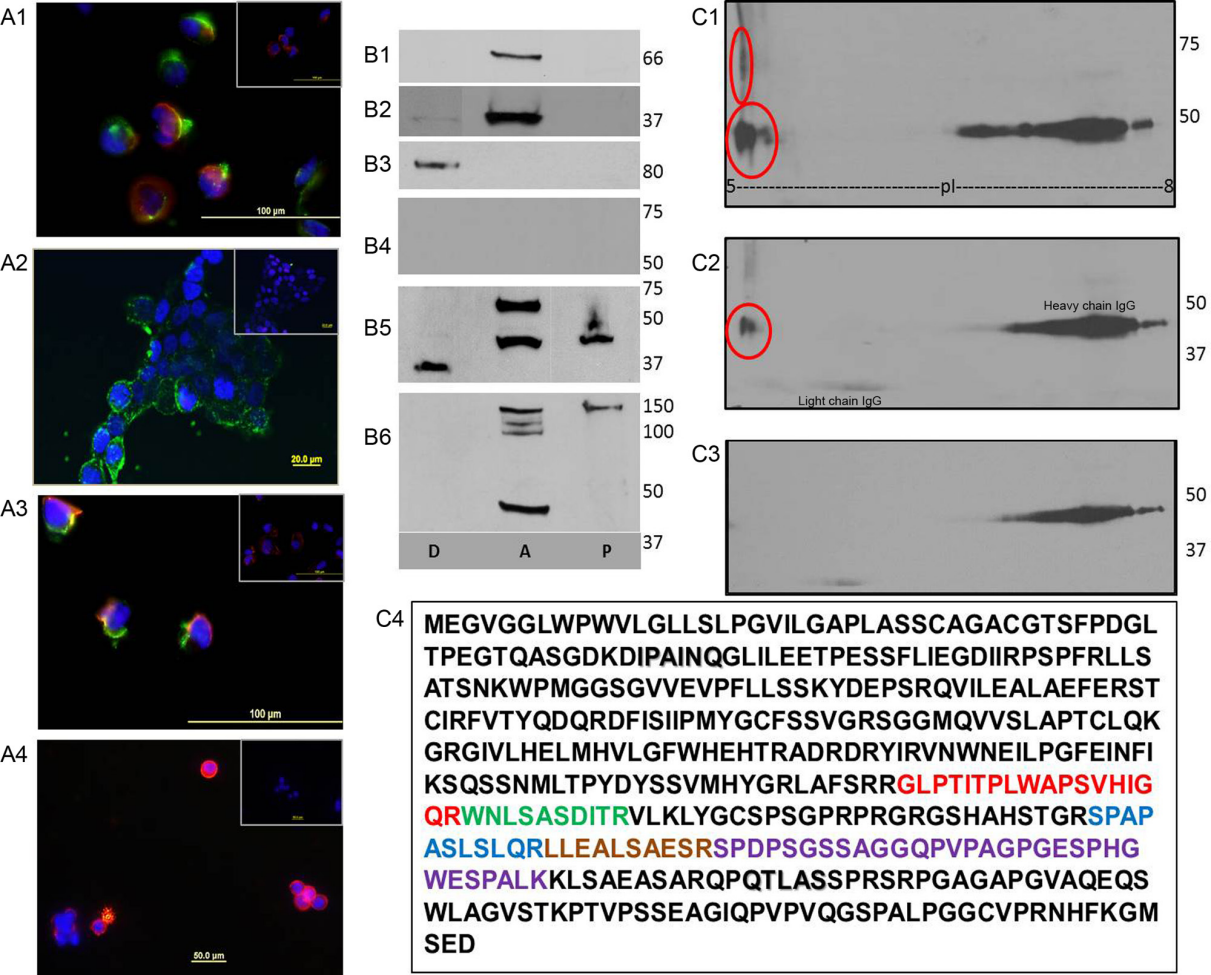
Membrane associated SAS1B in primary endometrioid cells and MMMT cell lines **Panel A:** Live cell indirect immunofluorescence: Using IM antibodies, surface SAS1B green signals are seen in S08-38710 (A1), SNU539 (A2), luciferase expressing SNU539 (A3); red SAS1B signal is present in primary endometrioid tumor cells recovered from a patient (A4). Respective insets show no staining using PIM antibody. **Panel B:** Results of phase partitioning of SAS1B isoforms extracted with TX-114 from SNU539 cells where D: detergent phase; A: aqueous phase; P: pellet. Partitioning of various control proteins such as albumin in A phase (B1), GAPDH in D and A phases (B2), and CD44 in D phase (B3) was observed in their expected fractions. The PIM antibody stained no proteins (B4). The IM antibody showed the presence of the ∼36–37 kDa form predominantly in the D phase, the ∼65 kDa form was localized in the A phase and the 46 kDa form was predominantly seen in the A phase with a relatively small portion in the D phase. Un-partitioned 46 kDa form also appeared in the P phase (B5). The PPpAb detected the 46 kDa form in the A phase along with several other higher molecular weight proteins in the A and P phases (B6). **Panel C:** Recovery of endogenous SAS1B microsequences by immunoprecipitation from SNU539 cells. Immunoprecipitated proteins were obtained using IM antibody, run on 2-D gels, and Western blotted with IM antibodies (C1), ASTL PP antibody (C2), or PIM sera (C3). The immunoreactive spots (red circles) were detected with both anti-SAS1B antibody reagents (C1 & c2) ranging between pI 5-6 while the immunoprecipitate stained with the PIM antibody did not contain detectable SAS1B proteins (C3). The trail in the right side of the blots is the heavy chain of IgG detected by the secondary antibody. A duplicate immunoprecipitate was sent for mass spectrometry and the ASTL/SAS1B peptides shown in various colors were recovered (C4), confirming reactivity of the IM antibodies with endogenous SAS1B.

Fibronectin-adhered uterine MMMT lines SNU539 and S08-38710 and control uterine line MAD10-252/616 were tested for SAS1B protein by IIF using IM and PIM antibodies. No immunostaining of any of the cell types was observed with PIM antibodies under the conditions of either live staining or cell permeabilization (Figures 3 and 4). When IM antibody was applied to fixed permeabilized cells, SAS1B localized throughout the cytoplasm and was concentrated in the perinuclear region in S08-38710 (Figure 3I) and SNU539 (Figure 3J) cells, while MAD10-252/616 cells (Figure 3K) were negative for SAS1B. In live cells stained with IM antibody a population of SAS1B (green stain) was localized on the tumor cell-surface in fibronectin-free S08-38710 (Figure 4A1) and SNU539 (Figure 4, 4A2 and 4A3) cells. Live stained cells obtained from primary endometrioid tumors by collagenase treatment followed by recovery for 2–3 hours in culture showed cell surface localization of SAS1B (Figure 4, 4A4, red immunofluorescence). In contrast, MAD10-252/616 cell preparations showed no surface immunostaining (Supplementary Figure S3).

### Phase partitioning identifies a putative cell surface SAS1B isoform

SNU539 cells were subjected to TX-114 extraction and phase partitioning, and partitioned fractions were analyzed by Western blotting with SAS1B antibodies. Specific biomarkers were employed to validate that the hydrophobic detergent (D) and hydrophilic aqueous (A) fractions were correctly isolated. Positive control albumin at 66 kDa, a known hydrophilic protein, partitioned in the A phase (Figure 4B1). GAPDH partitioned in the A phase although a minor amount partitioned in the D phase (Figure 4B2) (The subcellular localization of GAPDH is known to be principally cytosolic but membrane forms have been reported). The known membrane biomarker CD44 partitioned only in the D phase (Figure 4B3). Comparisons of the SAS1B protein banding patterns in different fractions revealed that immunoreactive SAS1B isoforms partitioned in both detergent (D) and aqueous (A) phases and in the residual cellular pellets (P) following phase separation (Figure 4B5). Use of the SAS1B IM antibodies showed the D phase was particularly enriched in the ∼36–37 kDa isoform, while the 46 kDa form was faintly seen in the D phase with the majority being concentrated in the A phase along with the ∼65 kDa SAS1B isoform. Using the PPpAb antibody, the 46 kDa isoform was also identified in the A phase along with other higher molecular weight proteins (Figure 4B6). Together, these results indicate that SAS1B isoforms partition in TX-114 into both D and A fractions and suggest that the ∼36–37 kDa SAS1B isoform in the hydrophobic fraction (D) may represent a dominant form of the membrane species.

### Immunoprecipitation with anti-SAS1B IM antibody recovered SAS1B protein microsequences

Endogenous SAS1B was immunoprecipitated by IM antibody from SNU539 cells and analyzed by 2-D SDS PAGE Western blots. The dominant 46 as well as the ∼65 kDa SAS1B isoforms were recovered (Figure 4C1, red circles). A parallel blot was stained with the PPpAb antibody and detected the 46 kDa isoform (Figure 4C2). No immunoreactivity with the PPpAb to SAS1B was detected in immunoprecipitates stained with PIM antibodies (Figure 4C3) other than the heavy and light chains detected by the secondary antibody. Parallel SAS1B immunoprecipitates obtained with the IM antibodies in solution were analyzed by MS/MS to identify SAS1B peptides. The instrument was calibrated on peptides identified in a control digest of recombinant SAS1B. The data were analyzed by performing selected ion chromatograms (SICs) of 3 major fragments for each of the above MS/MS parents. Of the 6 unique peptide sequences that were available from the control digest of recombinant SAS1B, 5 SAS1B peptides were identified as perfect matches in the MS/MS spectra of the immunoprecipitate, thereby confirming the presence of SAS1B in the sample recovered from uterine cancer cells (Figure 4C4).

### Complement dependent cytotoxicity (CDC) of MMMT cells bearing surface SAS1B

**Figure 5:**
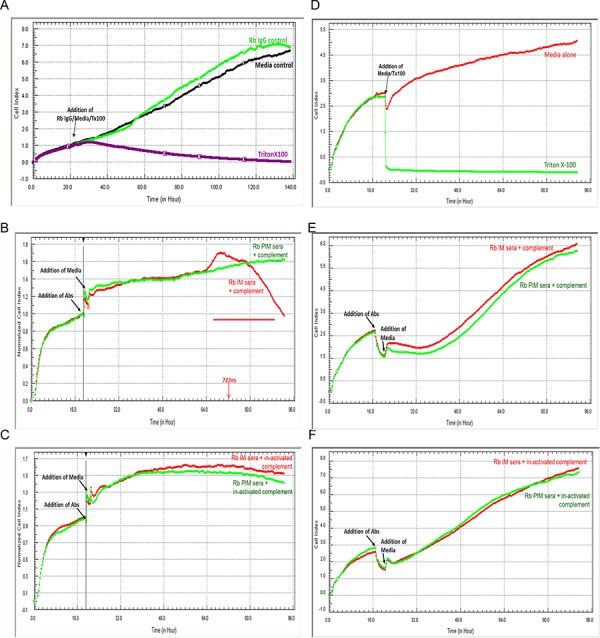
Complement mediated cytotoxicity with anti-SAS1B antibodies Cells grown on impedance electrodes were used to study the effects of antibodies and complement on cell growth. Panels A–C show SNU539 cells, while D–F depict MAD10 cells. **Panels A and D:** These panels show effects of media alone, media with a control rabbit IgG, and Triton X-100 detergent on impedance. Cells incubated with detergent lost contact with electrodes resulting in decreased impedance thus indicating growth arrest. **Panels B and E:** These panels show treatment with IM and PIM antibodies in the presence of active rabbit complement proteins. SNU539 cells (B) lost contact with electrodes and showed a drop in impedance upon treatment with IM but not with PIM antibodies, while no such effects were seen with MAD10 cells (E). When the complement proteins were deactivated by heat in-activation at 56°C, no decreases in impedance were observed with the IM antibodies on SNU539 cells **(Panel C)** and MAD10 cells **(Panel F).** PIM antibodies in all conditions showed no decreases in impedance.

The Xcelligence impedance electrode system was used to evaluate the effect of complement and anti-SAS1B antibodies on cell growth. Figure 5 shows SNU539 (5A) and MAD10 cells (5D), respectively, treated with control media and media supplemented with rabbit IgG, where impedance increased as cells grew. Wells treated with media containing 0.01% v/v Triton X-100 to impede growth showed a marked decrease in impedance. In the presence of active rabbit complement proteins, SNU539 cancer cells exposed to IM antibodies showed cytotoxic effects around 72 hours as indicated by a decrease in impedance (5B), whereas cells exposed to PIM antibodies and active complement showed no decrease (5B). Upon addition of complement proteins that had been heat in-activated, no decrease in impedance was seen as SNU539 cell growth continued (5C) in the presence of either IM or PIM antibodies (5C). The MAD10 cell line demonstrated no changes in impedance in the presence of active (5E) or inactive (5F) rabbit complement protein with either the IM or the PIM antibodies.

### After antibody binding membrane associated SAS1B was internalized in vesicles that entered the early and late arms of the endocytic pathway

**Figure 6:**
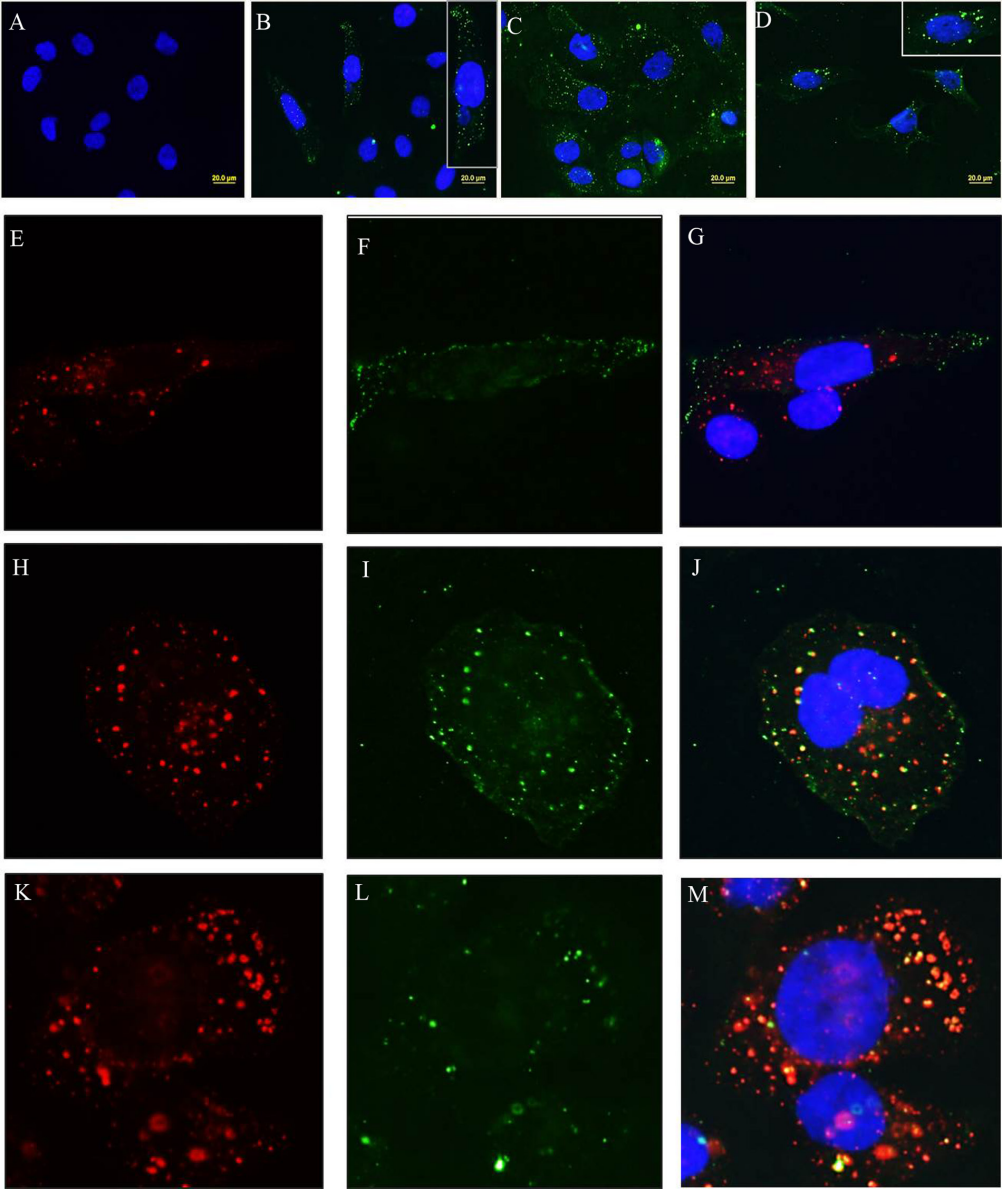
SAS1B-antibody complexes undergo endocytosis and co-localize with classical endocytic markers At various time points after SAS1B antibody treatment cells were studied to evaluate intracellular entry and co-localization with markers of classical endocytic vesicles and lysosomes. **Panel A:** This panel shows no internalization with control PIM antibodies after 60 min, while IM antibodies at 15 min **(Panel B)**, 30 min **(Panel C)** and 60 min **(Panel D)** show green immunofluorescent vesicles representing internalization of SAS1B-antibody complexes. Insets in B and D show higher magnifications. Punctuate green SAS1B+ endosomes were evident just beneath the cell membrane after 15 min of incubation at 37°C **(Panel F)** and did not co-localize with red EEA1+ vesicles **(Panel E** and merged in **Panel G)**. As incubation periods increased SAS1B+ vesicles coalesced into larger vesicles deeper within the cytoplasm **(Panels I** and **L)**. These larger SAS1B+ vesicles co-localized with red EEA1+ vesicles **(Panel H** and merged in **Panel J)** at 30 min and with red LAMP1+ vesicles **(Panel K** and merged in **Panel M)** at 60 min.

SAS1B IM and PIM antibodies were used in immunofluorescence experiments to test if SAS1B was internalized after antibody complexing and, if so, to determine the temporal pattern of internalization (Figure 6). At intervals of 15, 30 & 60 min after immune-complex formation, intracellular entry and SAS1B co-localization were studied using classical markers for endocytic vesicles and lysosomes. Punctuate green SAS1B^pos^ endosomes were evident just beneath the cell membrane (6B) after 15 min incubation at 37°C with the IM antibody. The vesicles were more numerous and were deeper in the cytoplasm at 30 min (6C), and they appeared to have coalesced into larger vesicles deep within the cytoplasm by 60 min (6D). The small endocytic vesicles comprised a distinct population just beneath the plasma membrane at 15 min (6F & G) but as time progressed the larger SAS1B^pos^ vesicles co-localized with red EEA1^pos^ endosomes (6J) at 30 min and with red LAMP1pos endosomes (6M) at 60 min. Control PIM at all time periods (e.g. at 60 min in panel A) revealed no internalization.

### Growth of MMMT cells was arrested with an immunotoxin targeted to SAS1B

**Figure 7:**
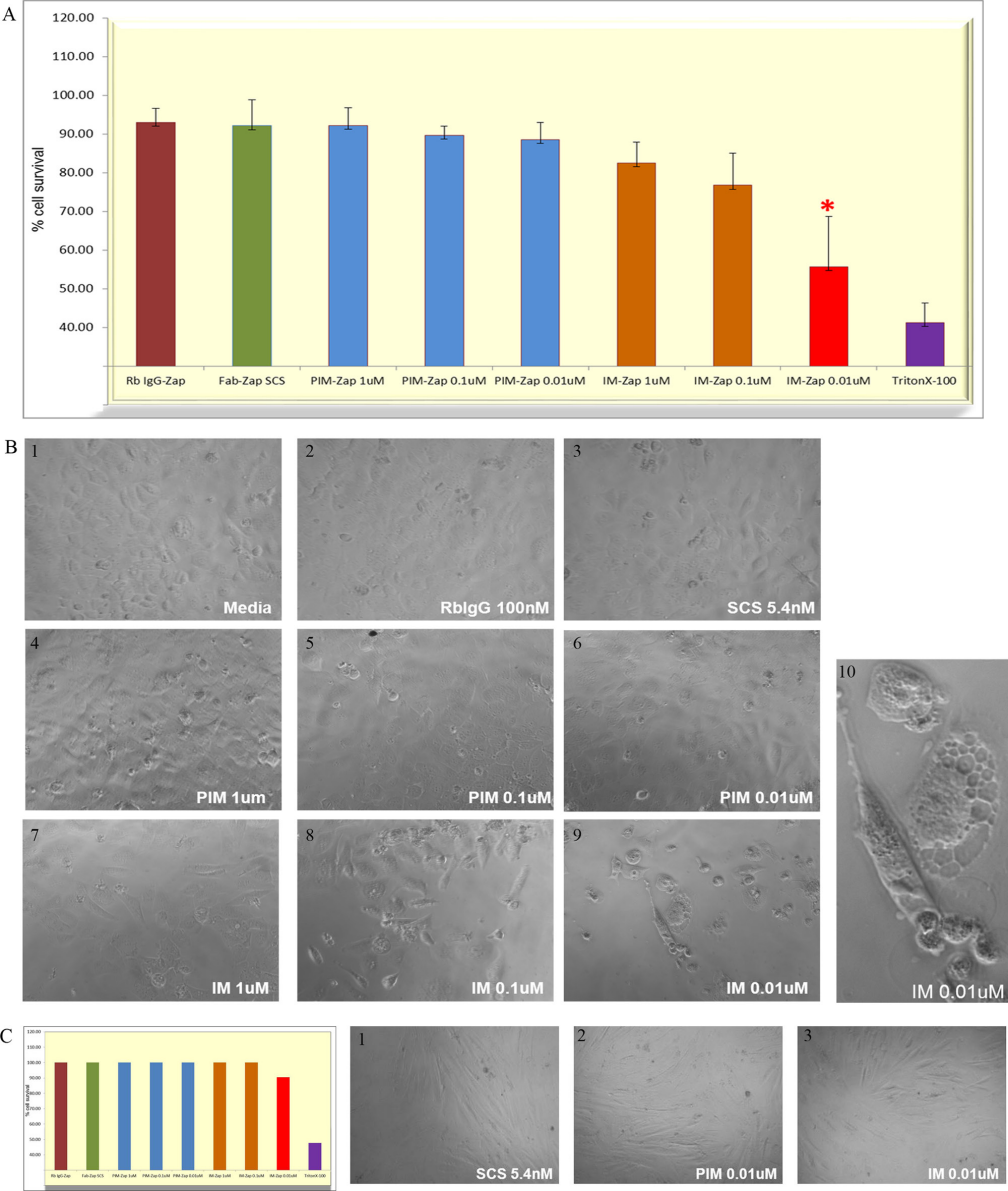
Arrest of SNU539 cell growth with a saporin-immunotoxin targeting SAS1B Cells in culture were exposed to varying concentrations of IM and PIM antibodies complexed with a fixed concentration of 5.42 nM secondary saporin immunoconjugate and monitored over 72 hours. Cells were observed by light microscopy and assayed for total biomass; culture supernatants were assayed for LDH levels. **Panel A:** This shows mean percent survival of SNU539 cells in the presence of IM antibody saporin conjugates (*N* = 4 experiments). IM antibody at concentrations from 1 μM to 1 nM was used and concentrations of 1–10 nM showed significant inhibitory effects (7A and 7B) on growth while PIM antibodies at identical concentrations did not (blue bars 7A). Triton X-100 detergent was used as positive control to arrest growth at the outset of the treatment period (purple bar 7A). Normal rabbit IgG saporin, saporin conjugate alone (SCS), or media alone did not demonstrate growth arrest (7A). **Panel B:** Deleterious effects on cells noted by light microscopy include cell vacuolation, cell rounding, pyknosis, and death (7B9, magnified in 7B10). **Panel C:** Under identical conditions SAS1Bneg MAD10 cells did not exhibit growth arrest in culture (7C) and MAD10 cells did not demonstrate deleterious microscopic effects after similar treatments (Panel 7C1–7C3).

An indirect immunotoxin assay was employed to evaluate tumor cell growth after treatment with SAS1B IM and PIM primary antibodies and a secondary saporin-immunoconjugate (SCS) [21] by assessing in-vitro growth with the sulforhodamine B assay [22]. During the 72 hours culture with SAS1B IM antibodies the growth of SNU539 cells was inhibited (Figure 7A, red bars) compared to that of cultures exposed to PIM antibodies (Figure 7A, blue bars). The SCS was kept constant at 5.42 nM and the cell killing effect was evident at concentrations of 1–100 nM IM antibody (Supplementary Figure S4). This assay showed a classic prozone effect (Hook effect) at high primary antibody concentrations, when target cell surface SAS1B was saturated with unlabeled antibody. At concentrations of 0.01–0.001 uM, near the stoichiometry of the secondary Fab-Zap conjugate (5.42 nM), growth arrest and killing were striking (Supplementary Figure S4). By contrast, growth of SAS1B^neg^ MAD10 cells was not impeded by identical concentrations of IM or PIM antibodies (Figure 7C). Additional control cultures showed no cell killing, including cells treated with media alone, the SCS alone, and a normal rabbit IgG conjugated with saporin (Figure 7A/7B). Treatment of the cultures with Triton X-100 to induce cell death at the outset of the incubation period was used as a positive control (Figure 7A). After the 3 days of SAS1B IM antibody treatment, SNU539 cells rounded, showed vacuolated cytoplasm, and had pycnotic nuclei compared to normal appearing cells treated with PIM antibody (Figure 7B, 9–10). Supernatants from cultures under all treatment conditions were also assessed for LDH activity as a marker of apoptosis. Supernatants from cultures exhibiting high growth arrest demonstrated significantly higher LDH levels compared to the controls, suggesting that cell death via apoptosis occurred in those samples in which growth arrest was detected (Supplementary Figure S5).

## DISCUSSION

### SAS1B is a novel tumor surface target in endometrioid and MMMT uterine cancers

Six lines of evidence support the candidacy of SAS1B as a novel tumor biomarker and drug target for an immunotherapeutic approach in uterine cancer. First, SAS1B is exposed on the surface of uterine cancer cells where it is accessible to antibody binding. Second, antibodies in the presence of complement arrest the growth of SAS1B^pos^ uterine cancer cells. Third, after being bound by antibodies at the cell surface SAS1B internalizes into the endosomal-lysosomal system providing a pathway for drug internalization and payload release. Fourth, tumor cells expressing SAS1B can be killed by a SAS1B-directed immunotoxin that employs a pH sensitive linker arm and saporin payload. Fifth, SAS1B is expressed at high incidence in endometrioid and MMMT uterine tumors. Lastly, SAS1B's normal restriction among normal healthy tissues to the pool of growing oocytes in the ovary provides a strategy for tumor selective targeting in cancers that express this cell surface protein.

### SAS1B is accessible on the surfaces of tumor cells

SAS1B was detected in permeabilized ASTL mRNA^+^ tumor cells throughout the cytoplasm and was concentrated in the perinuclear endoplasmic reticulum/Golgi region. This observation is in concert with SAS1B translocation into the ER lumen as predicted from the presence of an N-terminus signal peptide on each of three ASTL splice variants in mice [1] and from the signal peptide encoded by exon 1 of the human NCBI reference sequence [NM_001002036]. In addition to this intracellular population, SAS1B molecules were also imaged by staining on the surfaces of live cells recovered from both primary uterine tumors and established MMMT cell lines. Western blot analysis of the SNU539 extract reveals unique forms of the protein; an expected 46 kDa form that was also identified in the human ovary total extract and 2 other forms viz., a 65 kDa form, a potential isoform unique to tumor cells and a 36–37 kDa form likely the active membrane form of this metalloproteinase deduced from losing the signal as well as pro-peptide domains. The detection of a population of SAS1B accessible to antibodies on the surface of uterine tumor cells supports the concept that SAS1B can be targeted by antibodies and antibody-drugs (Figures 3 and 4).

### Complement dependent cytotoxicity (CDC)

In the presence of active complement SAS1B IM antibody induced growth arrest around 72 hours, while all controls including PIM antibody and heat inactivated complement showed continued cell growth. The demonstration that antibodies to SAS1B can induce CDC further supports the surface accessibility of SAS1B and its candidacy as an immunotherapeutic target and suggest that production of complement-fixing monoclonal antibodies directed to the SAS1B ectodomain should be considered an important future goal for a “naked” (stand-alone) monoclonal antibody approach to targeted immunotherapy.

### SAS1B internalizes into the endocytic pathway in uterine tumor cells after antibody binding

A key goal in target validation for cancer immunotherapy is evaluation of receptor-mediated endocytic pathways as routes for intracellular drug delivery. Subsequent to ligand binding cell membrane proteins may internalize through one or more entry portals including clathrin-coated vesicles, caveoli, and lipid rafts [23, 24]. Changes in vesicle microenvironments along the endolysosomal pathway have been exploited for releasing cytotoxic drugs linked to antibodies including: 1) addition of lysosomal hydrolases that catalyze breakdown of peptide, ester, or disulfide bonds; 2) increased redox potential due to higher concentrations of reduced glutathione and cysteine that can reduce disulfide bonds; and 3) the acidic pH of ∼5 in the lysosome [25]. Chemistries using acid-labile hydrazone linkers, sterically hindered disulfide linkers, and peptide linker arms have been designed to take advantage of the endolysosomal microenvironment for drug release. Since internalized vesicles of all three entry pathways may converge into the early endosome and eventually end at the lysosomes, at various time points after restoration of membrane fluidity SAS1B was co-localized with EEA1, a marker of the early endosome, and with LAMP1, a marker of late endosome. SAS1B-Ab complexes were internalized in SAS1B^pos^ cells within 15 minutes and displayed discrete sub-plasmalemmal localization in endosomal vesicles. The small endosomes coalesced with the early endocytic compartment (EEA1) at 15–30 min, and as time progressed (to ∼30–60 min) merged with the late endosomal pathway (LAMP1). A recent study with HER2-positive small-cell lung cancer cells demonstrated that the cell surface-binding of trastuzumab diminished with time during incubation at 37°C and was internalized, while most trastuzumab did not internalize and remained on the cell surface when incubated at 4°C without warming to 37°C [26]. Our studies also indicate that a SAS1B antibody-drug conjugate will reach the endolysosomal compartment once internalized, and it strongly supports SAS1B as a candidate for an immunotherapeutic approach using payloads conjugated with linker arms sensitive to the lysosomal environment (Figure 6).

### Uterine tumor cell growth is arrested with an immunotoxin introduced by targeting SAS1B

Cessation of uterine tumor cell proliferation was noted after delivery of a saporin toxin coupled to a secondary antibody that bound to SAS1B primary antibodies. The saporin toxin payload was linked to the secondary antibody via a proprietary pH sensitive linker arm that facilitated release of the ribosome-inactivating saporin protein in the acid environment of the lysosome [27], thus allowing it to affect protein synthesis and cause cell death via apoptosis (Figure 7). This demonstration opens the way for additional strategies for passive therapeutic antibodies that target SAS1B, including the use of various linker arms that are labile within the lysosomal compartment, and the testing of different drug payloads.

### SAS1B incidence in endometrioid and MMMT uterine cancers

The frequency of SAS1B expression overall was approximately 76.7% with the incidence in endometrioid cancers being slightly lower (66%) and in the more aggressive MMMTs somewhat higher (85%). These results underscore the importance of considering a theranostic paradigm in which SAS1B applications as a therapeutic candidate and diagnostic target are co-developed, with companion assays to identify SAS1B^pos^ tumors driving the selection and stratification of patients to receive targeted immunotherapy. Knowledge of the frequencies of SAS1B occurrence in uterine tumors will likely become more refined as additional tumor samples are examined for SAS1B/ASTL translation and transcription, but the trend is sufficiently clear from the exploratory cohorts to compare and contrast the pattern and frequency of SAS1B expression to established tumor surface targets.

### Unique attributes of SAS1B set it apart from other tumor associated surface antigens as a tumor-selective target

The identification and validation of novel tumor cell surface antigens comprises one of the most active and promising arenas in contemporary immunotherapeutic research [19]. Proteins displayed on the surfaces of tumor cells are attractive molecules for the inhibition of key signaling pathways by “naked” stand-alone monoclonal antibodies [28] or for selective delivery of cytotoxic molecules via monoclonal antibody-drug conjugates [18, 29]. Mechanisms of therapeutic action for these biological drugs may include producing programmed cell death, blocking growth factor receptors, arresting proliferation of tumor cells, inducing antibody-mediated cell killing via antibody-dependent cell-mediated cytotoxicity (ADCC), complement-dependent cytotoxicity (CDC), or the delivery of cytotoxic payloads that act to truncate DNA synthesis or inhibit microtubules [30]. Immunotherapy using monoclonal antibodies holds considerable promise as an anti-cancer therapeutic strategy because of its ability to target cancer cells specifically while sparing surrounding normal untransformed tissue [31]. Building on the completion of human genome sequencing more than a decade ago [32] the search for novel tumor associated surface antigens with improved properties constitutes a major area of opportunity to optimize the safety and efficacy of immunotherapeutics [33].

The current FDA approved immunotherapeutics for various cancer indications target molecules found on cancer cell surfaces but also on the surfaces of various types of normal healthy adult cells, resulting in a variety of on-target/off-tumor effects [34]. The human epidermal growth factor receptor 2 [Her2/neu], for example, found on a subset (20–30%) of breast tumors [35], is a target for Trastuzumab which, due to Her2's wide expression in human tissues including the adult myocardium, has a variety of adverse drug side effects including instances of cardiomyopathy [36]. CD30, a tumor necrosis factor (TNF) receptor targeted by Brentuximab, is highly expressed on Hodgkin's lymphomas and systemic anaplastic large-cell lymphomas; although this target's expression is restricted to the immune system, CD30 is also present on normal activated T, B, NK cells, and monocytes [34]. Thus, adverse drug effects of immunotherapeutics targeting CD30 include neutropenia and sepsis. One promising therapeutic target is the interleukin (IL)-13 receptor α2 [31], which is expressed at high frequency in the aggressive and incurable form of primary brain tumor known as glioblastoma multiforme [37–39] and in other solid tumors [40], while normal tissues express little to no IL-13 Rα2 except for the testes [41].

The SAS1B metalloproteinase is unlike any other tumor surface antigen proposed as a candidate for immunotherapeutic strategies for cancer in that it offers a unique opportunity for selective tumor targeting. Most oncology drug targets (e.g., Her2, EGFR) exhibit a quantitative expression difference between tumor and normal tissue. In contrast, SAS1B has a qualitative and absolute expression difference between tumor and normal cells, with the exception of the pool of growing oocytes. Among normal adult human tissues SAS1B translation (Figure 1) is ovary and oocyte specific, appearing in primary-secondary transition follicles, while being absent in the ovarian reserve of primordial follicles. This stage specific translation of SAS1B in human ovaries is identical to its expression pattern in other eutherians [2]. Live stained fertilized human oocytes display a population of SAS1B molecules accessible to antibody binding on the oolemmal surface where SAS1B appears uniformly distributed. It is important to note that SAS1B protein is not expressed in stem cells of the gastrointestinal tract and ASTL messages are absent in human derived induced pluripotent stem cells (Supplementary Figures S6 and S7 respectively). SAS1B protein is not expressed in a variety of normal human tissues dotted in a tissue microarray and tested by IHC using the IM antibodies (Supplementary Figure S8) and no ASTL ESTs have been deposited in NCBI Unigene database from normal tissues (Supplementary Figure S9). These observations suggest that an immunotherapeutic agent targeting SAS1B^pos^ tumors may have on-target/off-tumor effects in the ovary on the pool of growing oocytes in developing follicles, but on-target/off tumor effects would be absent in other normal organs. The chronology of folliculogenesis in women indicates that ≥300 days may be required from initial recruitment of primordial follicles through ovulation [42, 20]. Although the exact time period required for oocytes to ovulate following initiation of the second granulosa cell layer (primary-secondary follicle transition) and the exact time during folliculogenesis when a population of SAS1B becomes accessible on the oocyte surface are not precisely determined in women, 300 days may be a reasonable estimate of the period required for restoration of fertility after administration of a SAS1B targeted immunotherapeutic. If used as a stand-alone front line treatment, because of SAS1B's absence in the pool of primordial follicles, such an agent might find particular acceptance in pre-menopausal cancer patients who have continued interest in child bearing.

### Cancer-oocyte antigens: an emerging research field paralleling that of cancer-testis antigens

To date, SAS1B has been proposed to function in blastocyst hatching [43], sperm-egg interaction [1], the block to polyspermy [3], epithelial cell behaviour during early development in chick [44], and as a secretory protein released by oocyte activation [45]. Previously proposed translational applications of ASTL include its use as an ovarian cyst fluid biomarker [46] and target for an oocyte-specific contraceptive [2]. Since the identification and cloning of the first cancer-testis antigen [47] and LJ Old's proposal that reactivation of male gametogenic expression programs is involved in the malignant transformation process [48–51], it has become evident that somatic cell-to-male germline dysregulation is a general hallmark of a wide range of human tumors. Reactivation of the male gamatogenic expression program is associated with highly malignant phenotypes and, along with a tumor environment characterized by hypoxia and inflammation, is thought to be limiting for conventional therapy [52, 53]. SAS1B is a pioneer drug target in a similar and emerging field termed cancer-oocyte antigens that parallels that of cancer-testis antigens. Cancer-oocyte antigens may be defined as molecules restricted to the female gametogenic pathway in the adult ovary that show aberrant expression in cancer tissues, with SAS1B's transcription and translation in uterine tumors, as demonstrated in this study, being prototypic. This unique expression profile in the pool of growing oocytes coupled to SAS1B's identification as a membrane associated protein in uterine tumor cells present an opportunity for development of selective tumor treatments which spare both normal tissues and the ovarian reserve. We are currently limited by an incomplete understanding of the role of SAS1B in tumor cell biology and if it plays a direct role in tumor progression and metastasis. Understanding the role of SAS1B in basic cancer biology, its splice variants and isoforms, and developing appropriate well characterized monoclonal antibodies for targeted therapy is of interest to us and is being actively pursued in our laboratory.

## MATERIALS AND METHODS

### Human samples

Tumor and control uterine biopsy samples were obtained from the Department of Obstetrics and Gynecology under the IRB-HSR #15128 or were obtained as processed cDNAs from Bioline, USA. Paraffin embedded zinc-formalin fixed human uterine tumor tissue microarrays were obtained from the Department of Pathology. Bouin's fixed normal human ovary sections were obtained from the Pathology laboratory of Antoine Beclere Hospital in Clamart, France. Two improperly fertilized (poly-pronuclear) eggs donated under IRB #MJH 06-017 by patients undergoing *in vitro* fertilization treatment were obtained from Martha Jefferson Hospital in Charlottesville. Tissues for deriving cell lines were obtained from the University of Virginia Biorepository and Tissue Procurement Facility.

### Antibodies and other reagents

Rabbit anti-SAS1B polyclonal antibodies (IM) and control pre-immune serum (PIM) [2] were used either as purified IgGs (Mellon IgG purification kit, Pierce, USA) or as diluted sera along with rabbit pro-peptide ASTL polyclonal antibody (PPpAb) (#ab59889 Abcam, Cambridge, MA). Fab’-specific peroxidase labeled secondary antibodies (Jackson Immunoresearch, USA) were used for immunohistochemistry (IHC), Western blotting, and immunoprecipitation. For indirect immunofluorescence (IIF) anti-rabbit Alexafluor conjugates (Molecular Probes, USA) were employed.

### Tissue processing for RNA, cDNA and proteins

Tumor biopsy tissues were used for histology and RNA or total protein extraction. RNA was extracted using a Qiagen kit (with DNase digestion) and cDNA was synthesized using the Promega Improm kit. Proteins were isolated using Celis buffer and estimated with Bradford's Coomassie reagent (Pierce, USA). Tissues were processed for IHC as described earlier [2].

### Immunohistochemistry

Briefly, sections were melted, deparaffinized, quenched in methanol-hydrogen peroxide, rehydrated [54, 55] followed by antigen retrieval (Vector Labs, USA), and blocked with 5% non-fat dry milk (NFDM) containing 5% normal goat serum in PBS (NGS) for 1 hour at room temperature. A 1:100 dilution or 2 μg/ml concentration of IM or PIM antibodies was applied to slides at 4°C overnight. Following three washes, a 1:500 dilution of GαRb HRP was added. After additional washings, brown reaction product was developed using 3, 3′-diaminobenzidine (SIGMA, USA), followed by hematoxylin counterstaining and imaged after mounting.

### Cell culture

Cell culture conditions for uterine MMMT-derived SNU539 (fast growing, stable secondary cell line), a gift to co-author Dr. Hui Li through Dr. Park at the Seoul National University [56]; S08-38710 (very slow growing, primary carcinosarcoma cell line); and MAD10-252/616, hTERT immortalized postmenopausal non-cancer endometrial derived control cell line, are described in the supplementary section.

### Primers and RTPCR

Primers (Invitrogen, USA) were designed to specifically amplify ASTL (NCBI gene accession number NM_001002036) among the 134 zinc metalloproteases in the human genome. N-terminus primers: Fw-5′-GCGCCCCTGGCCTCCAGCTGCGCA-3′ and Rv-5′- CACGACACCACTACCACCCATGGG-3′; C-terminus primers: Fw-5′-GGCTGCAGCCCAAGTGGCCCCAGG-3′ and Rv-5′-AGCAACACCGGGGGCACCTGCTCC-3′; catalytic domain primers: Fw-5′-GAGGTCCCCTT CCTGCTCTCCAGC-3′ and Rv-5′-GGCATGGGACCC TCTCCCACGGGG-3′ yielded amplimers of 237, 309, 579 base pair respectively. For PCR, AmpliTaq gold 360 buffer kit was utilized (# 4398853, Applied Biosystems, USA).

### Western blotting

Harvested cells were lysed in Celis buffer containing protease inhibitor cocktail [57]. Proteins were electrophoresed and following transfer were blocked with NFDM-PBS and incubated with a 1:1000 or 5 μg/ml concentration of IM/PIM antibodies overnight at 4°C. After washes in PBS with 0.05% Tween-20 (PBST), blots were incubated with 1:5000 dilution of GαRb HRP for 1 hour, washed and immunoreactive bands were detected by ECL (GE Healthcare, UK).

### Immunoprecipitation of SAS1B protein, 2D gel electrophoresis and mass spectrometry

SAS1B was immunoprecipitated using IM antibodies. Antigen-antibody complexes were validated by 2D Western blotting [55] using IM/PIM and PPpAb antibodies. An independent immunoprecipitate was analyzed for ASTL peptides by mass spectrometry. Details are described in the Supplementary section.

### Phase partitioning of SAS1B protein isoforms

Two T300 flasks of SNU539 at 80% confluence were harvested with 2% precondensed Triton X-114 in Tris buffered saline (TBS) pH 7.4 on ice for 2 hours. Insoluble matter (P) was removed by centrifugation at 13,200 rpm for 15 min at 4°C. The protein supernatant was transferred to a clean chilled tube. Phase separation was done as follows: the tube containing the lysate was warmed at 37°C for 10 min and centrifuged at 13,200 rpm for 10 min at 25°C. A two phase preparation resulted: a clear micelle poor aqueous phase (A) at the top and an oily dense micellar rich detergent phase (D) at the bottom of the tube. Both phases were subjected to 4 cycles of phase separation in order to purify and enrich D and A preparations [58–61]. Both fractions were precipitated with acetone on ice. Precipitates were dried, solubilized in 2X Laemmli buffer, and heated at 99°C for 10 min. Equal amounts of D and A protein, along with the pellet fraction, were electrophoresed followed by Western blotting using one of several antibodies (at 1:1000 dilution): Rb pAb to BSA (Sigma, USA), 1:10, 000 dilution of mAb to GAPDH (Sigma, USA), mAb to CD44 (Cell Signaling, USA), PIM IgG, IM IgG, and PPpAb (Abcam, USA).

### Indirect immunofluorescence (IIF)

IIF on fixed cellsSNU539/ S08-38710 and MAD10-252/616 cells were grown on fibronectin coated coverslips (Sigma, USA), and 36 hours later, cells were fixed with 4% paraformaldehyde in DPBS (PFA) for 15 min at room temperature, washed with DPBS and blocked with 5% NGS. PIM/IM antibodies diluted 1:200 were added to coverslips and incubated at room temperature for 2 hours. Following washes, a 1:500 GαRb Alexa 488 secondary antibody containing DAPI and red phalloidin (to stain for actin filaments) was added for 1 hour in the dark, washed and mounted on slides with Slowfade (Molecular Probes, USA), dried, and imaged.

### IIF of living cells

Cells were grown on coverslips without fibronectin prior to incubation with primary antibody for 2 hours. Following media washes, cells were fixed in PFA and processed for subsequent steps as described above. Immunostaining was observed at 200–400X magnifications by fluorescent microscopy (Zeiss LSM 510-UV, Carl Zeiss, Germany). The same method was followed for the human oocyte SAS1B staining.

### Endocytosis assay

SNU539 cells were grown on coverslips, starved in serum free media for 2 hours and then incubated on ice for 20 min to immobilize the plasma membrane [62]. Cells were incubated on ice for 1 hour with IM or PIM antibody (1:100), washed three times with cold DPBS, and then either stopped at time 0 by fixing cells with 4% PFA or transferred to fresh prewarmed complete Roswell Park Memorial Institute medium (RPMI) at 37°C to allow antibody internalization to proceed for 5, 15, 30 or 60 min. The samples were fixed in 4% PFA, washed twice with DPBS and permeabilized with 0.1% Triton X-100 in PBS for 15 min. Next, cells were washed with DPBS and exposed to GαRb Alexa Fluor488 secondary antibodies diluted 1:500 for 60 min at 37°C. After final washes with DPBS, cells were mounted in slowfade with DAPI (Molecular Probes, OR, USA) and then imaged.

### Complement dependent cytotoxicity (CDC)

Twenty-five thousand SNU539 and control MAD10-252/616 cells were plated in single wells of 16 well eplates (Roche Diagnostics, USA), and incubated overnight in RPMI media containing heat-inactivated fetal bovine serum (FBS, Atlanta Biologicals, USA). Nineteen hours later, IM or PIM antibodies diluted tenfold were added to the wells and allowed to interact with cells for 3 hours. Inherent complement activity in the rabbit antisera was heat inactivated by heating at 56°C for 25 min. With careful handling, wells containing the cells and their respective antibodies received either 50 units of rabbit complement proteins/well (MP Medicals, USA) or 50 units of heat inactivated rabbit complement proteins/well. Cell growth was assessed with impedance (a correlate of the number of cells) using the xCELLigence system (Roche/ACEA Biosciences, USA); data were monitored over a period of 72–96 hours.

### Indirect saporin immunotoxin assay

SNU539 and MAD10 cells were dissociated using TrypLE (Invitrogen, USA), and allowed to recover for 1–2 hrs to replenish cell surface antigens prior to the assay (details discussed in Supplementary method section). A cell titration assay was first performed to monitor the cell density over 72 hours in order to avoid overcrowding in the wells during culture, and 4000 cells/well was selected as an optimal seeding concentration (data not shown). Cells were plated at a concentration of 4000 cells/200 μL heat inactivated media/well in triplicate in 96 well culture plates (Nalgene Nunc, Denmark). Cells were incubated overnight at 37°C in 5% CO_2_ to accommodate to the media after trypsinization. Next day, cells were incubated with premade immune complexes developed by co-incubation for 45 min of varying concentrations of primary antibody (1 μM, 0.1 μM, and 0.01 μM) and a fixed concentration of 5.42 nM of SCS (Advanced Targeting Systems, USA, Cat #IT-57). Controls consisted of media with SCS alone (negative control), direct tagged rabbit IgG-saporin at 100 nM concentration (direct conjugate negative control) (Advanced Targeting Systems, USA, Cat # IT-35), and 0.01% Triton X-100 (positive control) for cell lysis. After 72 hours incubation, cells in the experimental groups were imaged by light microscopy and then processed for bio mass estimation using the sulforhodamine B (SRB) assay. The SRB colorimetric assay is used for cell density determination, based on the measurement of cellular protein content. Absorbance readings are indicative of cell proliferation with lower absorbance indicating cell sloughing and cell lysis.

## SUPPLEMENTARY MATERIALS AND METHODS FIGURES


