# Correction: Membrane associated cancer-oocyte neoantigen SAS1B/ovastacin is a candidate immunotherapeutic target for uterine tumors

**DOI:** 10.18632/oncotarget.15755

**Published:** 2017-02-27

**Authors:** Eusebio S. Pires, Ryan S. D'Souza, Marisa A. Needham, Austin K. Herr, Amir A. Jazaeri, Hui Li, Mark H. Stoler, Kiley L. Anderson-Knapp, Theodore Thomas, Arabinda Mandal, Alain Gougeon, Charles J. Flickinger, David E. Bruns, Brian A. Pollok, John C. Herr

**Present:** The currently listed corresponding author, John C. Herr, is deceased. Oncotarget wishes to express our condolences to his family and colleagues.

Corrected: The new correspondence author information is as follows.

Original article: Oncotarget. 2015; 6(30):30194-211. doi: 10.18632/oncotarget.4734.

***Correspondence to:*** *Eusebio S. Pires,* ***email:*** *eusebiopires@gmail.com*

