# Supplementary material for: Membrane associated cancer-oocyte neoantigen SAS1B/ovastacin is a candidate immunotherapeutic target for uterine tumors
Source: Oncotarget. 2015 Aug 18;6(30):30194–211. doi: 10.18632/oncotarget.4734 (PMC4745790; doi:10.18632/oncotarget.4734)
Supplement: Supplementary file 1 [file oncotarget-06-30194-s001.pdf]

SUPPLEMENTARY MATERIALS AND METHODS

Cell lines

Cell culture grade reagents and media were procured from Invitrogen. The tissue biopsies were incubated in RPMI or Dulbecco's modified eagle medium (DMEM) supplemented with 1% Penicillin-streptomycin and 0.25 µg/ml fungizone for 3 hours at 4°C, rinsed in sterile PBS, and incubated in dispase I solution (0.26 mg/ml) overnight at 4°C. Exposed and loosely bound endometrial cells from the tissue were scraped with a surgical scalpel onto a culture container, cultured with a minimum volume of DMEM with high glucose containing 10% FBS (for MAD10-252/616), RPMI with L-glutamine, 10% FBS (for S08-38710), and 1% Penicillin-Streptomycin-Glutamine. The cultures were then left undisturbed for 3 to 4 days at 37°C in a humidified 5% CO_2 incubator. MAD10-252/616 cells were further immortalized by use of a retroviral vector that expresses hTERT and stable cells were selected by puromycin. SNU539 lines were maintained in the same media as S08-38710 lines. All media contained heat in-activated FBS.

Cell dissociation reagent: TrypLE™ Select (Invitrogen/Life Technologies, Catalog # 12563-011), was used to dissociate cells and like trypsin, it cleaves peptide bonds on the C-terminal sides of lysine and arginine. However, TrypLE™ Select's exceptional purity increases specificity due to the action of a single enzyme. This reduces damage caused by cleavage from multiple enzymes in trypsin preparations. Unlike porcine trypsin and other enzymes, TrypLE™ Select is free of animal-derived components, and is formulated on dedicated animal origin-free equipment. TrypLE select was found to be better than trypsin for passage of cells, resulting in increased clone survival [63]. Cells were allowed to recover for 1–2 hours following dissociation with this reagent prior to usage in an experiment.

Immunoprecipitation of SAS1B protein, 2D gel electrophoresis and mass spectrometry

One milligram of total SNU539 cell protein was incubated overnight with 8 ug IM antibody in quadruplicate. The following day, 50 µl of Protein-A Dynabeads (Invitrogen, catalog # 10006D) were added to the protein-antibody complexes and incubated at room temperature for 3 hours. Protein-A antigen-antibody complexes were washed and processed according to the manufacturer's protocol. Elution of immunoprecipitates from beads was carried out by resuspending washed bead complexes in 200 µl 2D gel rehydration buffer containing ampholines pH 5–8 and incubated for 2 hours on ice. 200 µl of eluted immunoprecipitated proteins were rehydrated on 11 cm ReadyStrip IPG strips (Bio Rad catalog # 163-2018) at a gradient of pH 5-8 and focused as described earlier [55]. Separation in the second dimension was performed using 11 cm IPG 8–16% polyacrylamide Criterion™ TGX™ Precast Gels (Bio Rad catalog # 567-1101). Gels were either transferred to nitrocellulose membrane for Western blotting, or silver stained for mass spectrometry analysis as previously described [2]. Membranes were blocked for 1 hour in 5% NFDM, 0.01% PBST, and 2% NGS, and probed overnight at 4°C with 3 µg/mL IM antibody, 1 µg/ µl PPpAb antibody (Abcam, catalog # ab59889), or 3 µg/mL PIM antibody. Blots were washed with PBST and incubated with a 1:5000 dilution of GαRb HRP, and immunostaining was detected by ECL. The remaining product of the IP was used for mass spectrometry analysis. The sample was diluted 8X with 100 mM AMBIC, reduced with 10 mM DTT and alkylated with 50 mM iodoacetamide (1 hour each). Then 0.5 µg of alkylated trypsin (Promega, USA) was added, and the digestion was allowed to proceed overnight at RT. The sample was made free of salts and reduced in volume with C18 trap columns. The resulting eluates were evaporated to 15 µL for MS analysis. The LC-MS system consisted of a Thermo Electron Orbitrap Velos ETD mass spectrometer system with a Protana nanospray ion source interfaced to a self-packed 8 cm × 75 µm id Phenomenex Jupiter 10 µm C18 reversed-phase capillary column. A 15 µL aliquot of the extract was injected and the peptides eluted from the column by an acetonitrile/0.1 M acetic acid gradient at a flow rate of 0.5 µL/min over 1.2 hours. The nanospray ion source was operated at 2.5 kV. The instrument was set to continuously do MS/MS on peptides identified in a control digest of SAS1B (563.9, 544.9, 981.2, 581.9, 838.6, and 648.7). The data were analyzed by selected ion chromatogram (SIC) of 3 major fragments for each of the above MS/MS parents.

Analysis of SAS1B in stem cell populations

Reverse Transcriptase PCR

RNAs from two induced pluripotent stem cell (iPSC) lines were gifted by Dr. Kun Bi, Sr. Staff Scientist R&D (Invitrogen- Life Technology). RNA was converted to cDNA using a Promega kit. PCR was carried out using 3 primer sets for 34 cycles using the Applied Bio Systems Gold Polymerase kit: GAPDH yielding a 210 base pair product; Oct4 stem cell marker 418 base pair product (Forward primer 5′-GGAAAGGCTTCCCCCTCAGGGAAAGG-3′; Reverse primer 5′-AAGAAC ATGTGTAAGCTGCG GCCC-3′); C-term SAS1B 309 base pair product. Products were run on 2% Agarose-TAE ethidium bromide gels.

IHC

Bouin's fixed Macaca *fascicularis* gastrointestinal tract tissue sections (stomach, duodenum, jejunum, bladder,

esophagus, salivary glands, and ovary) were obtained as a gift from Dr. Eliza Curnow (University of Washington in Seattle). Serial sections were processed as described earlier [2] and in the main text. Sections underwent antigen retrieval and followed by blocking in NGS-NFDM for 1 hour at room temperature. Sections were exposed to 1 µg in 200 µl of blocking media primary antibodies viz., SAS1B IM antibodies and rabbit anti LGR5 polyclonal (Proteintech # 21833-1-AP, USA) overnight in the cold room. The reaction product was detected as described earlier.

Cytoplasmic lactate dehydrogenase (LDH) assay

The cytotoxicity detection kit (LDH) from Roche Applied Science (Cat # 11644793001) was employed and followed the instructions provided. Briefly, after incubation for 72 hours, media from the wells of SNU539 cells containing the SAS1B-saporin conjugates were collected and centrifuged at 3000 rpm for 5 min to exclude (if any) free floating cells or sloughed cells. This was followed by aspiration of 100 µl of the spent media into a 96 well microtiter plate and addition of 100 µl of the LDH reaction mixture containing the catalyst and dye solutions. The mixture was incubated at room temperature for 30 min in dark. Prior to measuring the absorbance at 490 nm, 50 µl of stop solution was added and plate contents were mixed by gentle shaking.

SUPPLEMENTARY FIGURES

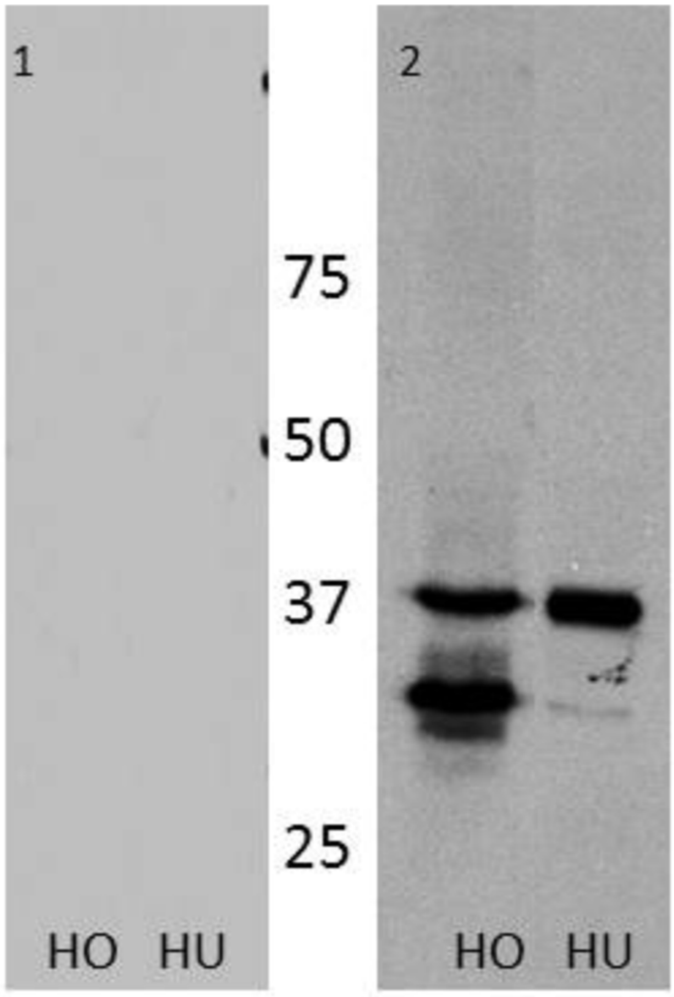

Supplementary Figure S1: PIM antibody and GAPDH control staining by Western blot analysis using total protein extracts from human ovary (HO) and uterine (HU) tissues. PIM antibody did not bind to any of the proteins in either the HO or the HU lanes (blot 1) indicating authenticity of staining with the IM antibody (shown in Figure 1F). Antibody to GAPDH (37 kDa protein mass) was used to demonstrate equal loading of proteins in ovary and uterine extracts. It is of interest to note that the immunodominant GAPDH isoforms at around 30 kDa are much more abundant in HO protein extracts than in the HU (blot 2).

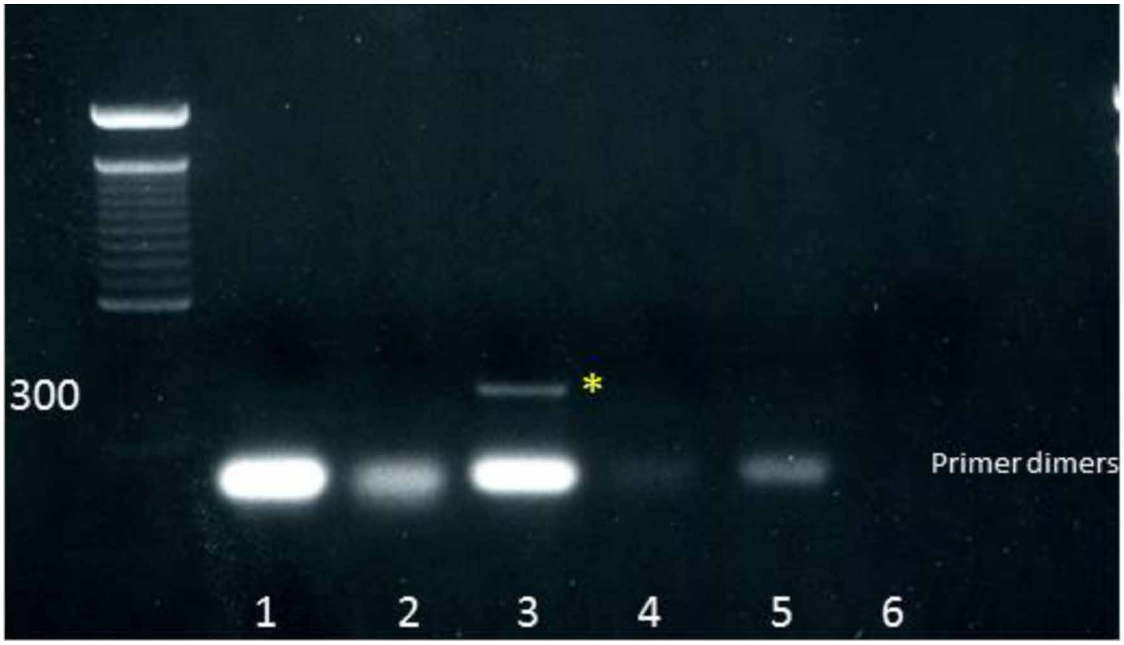

Supplementary Figure S2: Validation of SAS1B/ASTL PCR using gene specific primers. This PCR was performed to validate the 309 base amplicons obtained in all the PCR tests discussed in the manuscript. SNU539 cDNA was used as the source of the ASTL message. Lane 1: Master Mix + Polymerase – cDNA. Lane 2: Master Mix – Polymerase + cDNA. Lane 3: Master Mix + Polymerase + cDNA. Lane 4: Master Mix (Minus forward primer) + Polymerase + cDNA. Lane 5: Master Mix (minus reverse primer) + Polymerase + cDNA. Lane 6: gel loading dye. From the figure it can be appreciated that the 309 base pair ASTL amplicon was synthesized only in lane 3 which had all the PCR components, while all other lanes did not show amplification apart from the primer dimers that ran below 100 base pairs.

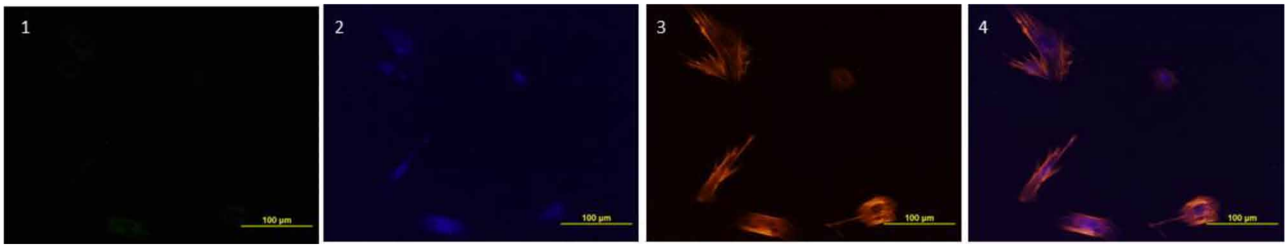

Supplementary Figure S3: SAS1B is absent on surface of live MAD10 cells by IIF using IM antibodies. IM antibodies were not immunoreactive with cell surface membrane proteins of live MAD10 control cells as indicated by the absence of green signals (Supplementary Figure S2 **Panels 1.** and **4.** which is a merge of 1–3). **Panel 2.** indicates DAPI blue nuclear counterstain and **Panel 3.** shows actin filament red staining by Phalloidin.

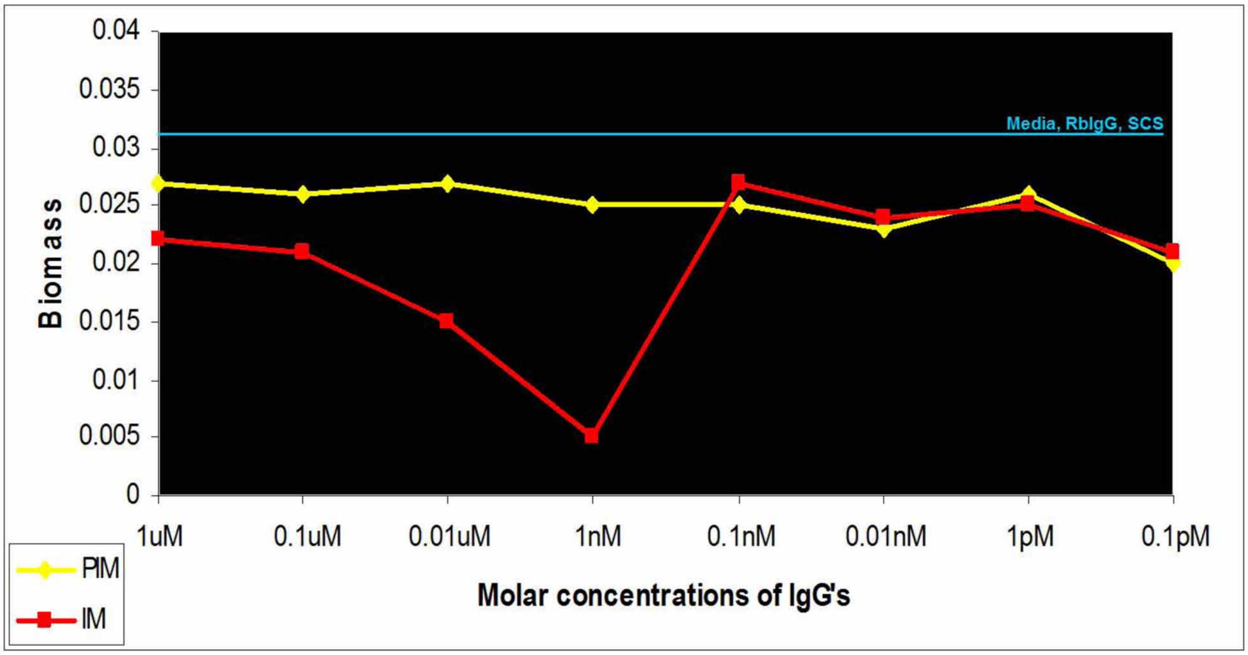

Supplementary Figure S4: Hook effect is seen with the IM antibody in the indirect saporin assay. The indirect saporin assay showed a classic prozone effect (Hook effect) at high primary antibody concentrations, when target cell surface SAS1B was saturated with unlabeled antibody. At concentrations of the IM antibodies (1–10 nM) spanning the stoichiometry of the secondary Fab-Zap conjugate (5.42 nM), growth arrest and killing were striking. Further dilution of the antibody did not show any killing activity.

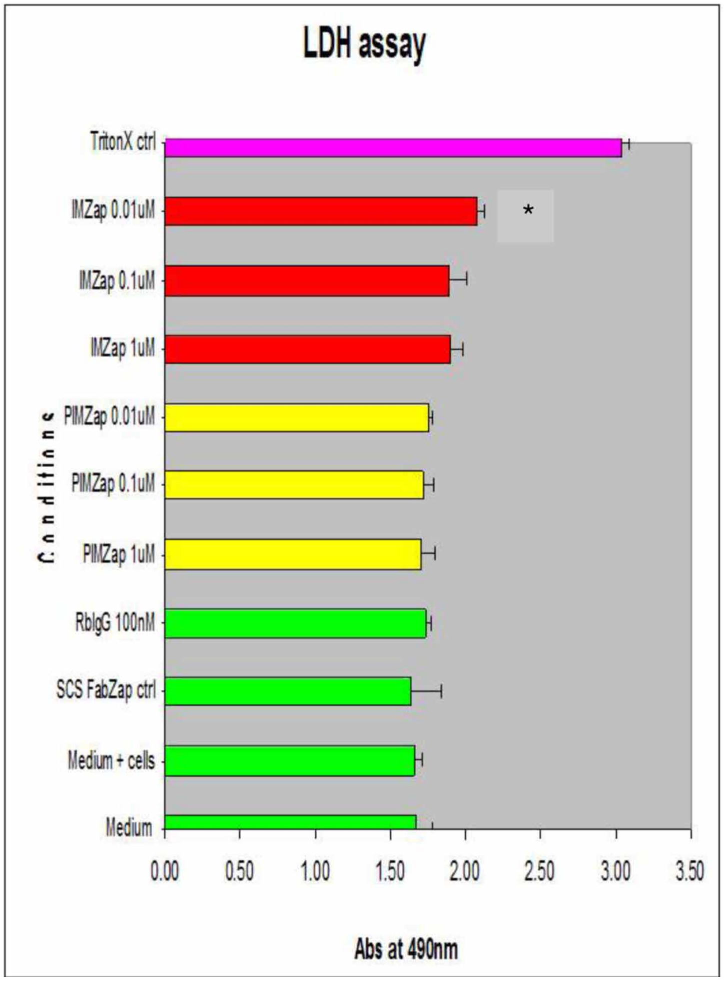

Supplementary Figure S5: LDH activity was observed in spent media from the indirect saporin assay. Figure shows levels of LDH assayed from debris-free spent culture supernatant collected from SNU539 experimental wells. Wells incubated with 0.01 µM IM-saporin conjugates [*] showed statistically significant increases in LDH levels (students *t*-test, $p = 0.002$) in comparison to the PIM at the identical concentration; thereby indicating that cell death occurred in the presence of IM antibody likely via apoptosis in those samples in which growth arrest was detected.

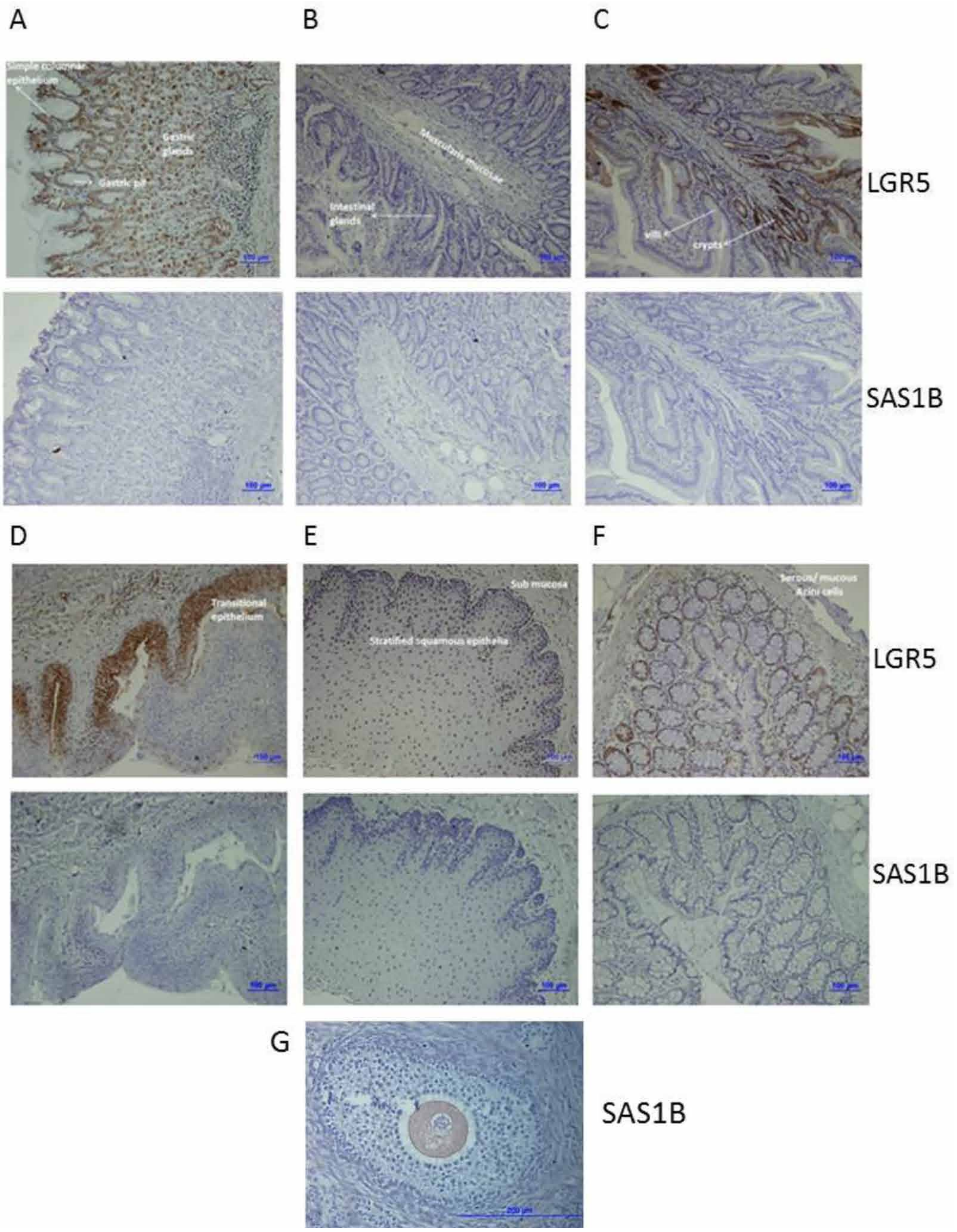

Supplementary Figure S6: SAS1B is absent in stem cells of the primate gastrointestinal tract tissue by IHC. Serial sections of monkey GI tract were stained with LGR5, a stem cell biomarker, or SAS1B IM antibodies in IHC. Tissues included stomach, duodenum, jejunum, bladder, esophagus, and salivary glands (**Panels A–F.** respectively). Except for duodenum all tissues stained for the GI stem cell biomarker LGR5. However, SAS1B was not immunoreactive in any cell type in any of these tissues, indicating that the LGR5^{pos} stem cell population does not express SAS1B. **Panel G.** is a positive control monkey ovary section stained with the IM antibody showing oocyte localization of SAS1B in an antral follicle.

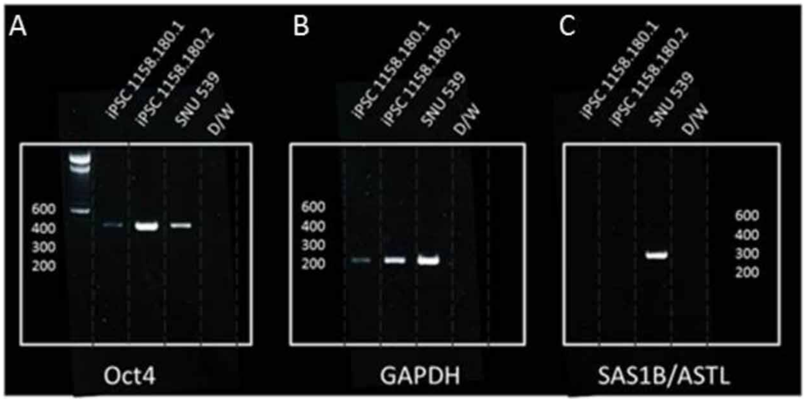

Supplementary Figure S7: SAS1B/ASTL is absent human induced pluripotent stem cells. Two iPSC cDNAs were tested by PCR for ASTL messages along with SNU539 (a positive control for SAS1B expression) and a distilled water (D/W) control. Stem cell marker Oct4 **A.** housekeeping GAPDH **B.** as well as C-term SAS1B **C.** primers were used. Oct4 expression was observed in both iPSC lines and interestingly in the SNU539 lane. However, SAS1B/ASTL expression was restricted to SNU539 uterine cancer cells and both iPSC lines did not show ASTL messages.

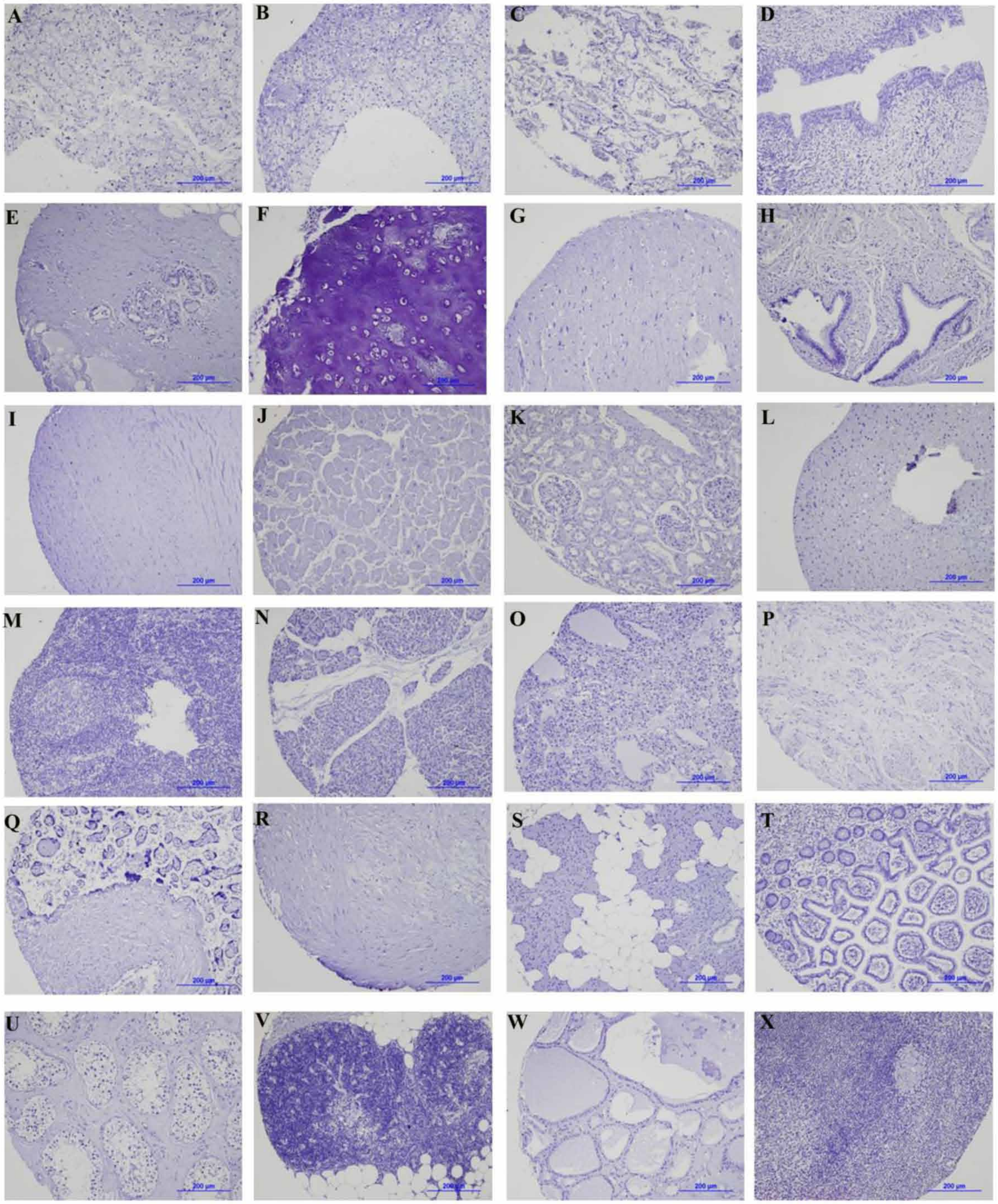

Supplementary Figure S8: SAS1B protein expression in normal human tissue microarray by IHC. IHC was performed on an array of PFA fixed; paraffin embedded normal human tissues using IM antibodies. As seen in the figure, no brown immunoreaction products are observed in any of the panels (**A.** adrenal cortex, **B.** adrenal medulla, **C.** alveoli, **D.** bladder, **E.** breast epithelium, **F.** cartilage, **G.** cerebral cortex, **H.** fallopian tube, **I.** heart aorta, **J.** heart myocardium, **K.** kidney cortex, **L.** liver, **M.** lymph node, **N.** pancreas, **O.** parathyroid, **P.** peripheral nerve, **Q.** placenta villi, **R.** prostate, **S.** salivary glands, **T.** small intestine mucosa, **U.** testes, **V.** thymus, **W.** thyroid and **X.** tonsil). Magnification at × 200. Positive control staining of human ovary is shown in Figure 1 of the main manuscript.

NCBI » UniGene » EST Profile Viewer

Pubmed Nucleotide Protein Genome Structure Popset Taxonomy

Search UniGene Go Clea

EST Profile

Hs.447993 - ASTL: Astacin-like metallo-endopeptidase (M12 family)

Breakdown by Body Sites

Pool name/ tissue	Transcripts per million	Spot intensity	Gene EST/Total EST in pool
			Hs.447993
adipose tissue	0		0 / 12866
adrenal gland	0		0 / 32940
ascites	0		0 / 39834
bladder	0		0 / 29860
blood	0		0 / 122252
bone	0		0 / 71618
bone marrow	0		0 / 48737
brain	0		0 / 1092688
cervix	0		0 / 48486
connective tissue	0		0 / 149072
ear	0		0 / 16100
embryonic tissue	0		0 / 212896
esophagus	0		0 / 20154
eye	0		0 / 208840
heart	0		0 / 89524
intestine	0		0 / 231981
kidney	0		0 / 210778
larynx	0		0 / 23466
liver	0		0 / 205291
lung	0		0 / 334815
lymph	0		0 / 44302
lymph node	0		0 / 89748
mammary gland	0		0 / 151230
mouth	0		0 / 66150
muscle	0		0 / 106371
nerve	0		0 / 15535
ovary	0		0 / 101488
pancreas	0		0 / 213440
parathyroid	0		0 / 20594
pharynx	0		0 / 40725
pituitary gland	0		0 / 16526
placenta	0		0 / 283019
prostate	0		0 / 189536
salivary gland	0		0 / 20265
skin	0		0 / 210759
spleen	0		0 / 53397
stomach	0		0 / 95679
testis	0		0 / 435204
thymus	0		0 / 79697
thyroid	0		0 / 46583
tonsil	0		0 / 17021
trachea	0		0 / 51780
umbilical cord	0		0 / 13764
uterus	4		1 / 232093
vascular	0		0 / 51649

Breakdown by Health State

Pool name/ tissue	Transcripts per million	Spot intensity	Gene EST/Total EST in pool
			Hs.447993
adrenal tumor	0		0 / 12655
bladder carcinoma	0		0 / 17584
breast (mammary gland) tumor	0		0 / 93090
cervical tumor	0		0 / 34484
chondrosarcoma	0		0 / 82838
colorectal tumor	0		0 / 112517
esophageal tumor	0		0 / 17245
gastrointestinal tumor	0		0 / 118498
germ cell tumor	0		0 / 263230
glioma	0		0 / 107194
head and neck tumor	0		0 / 133826
kidney tumor	0		0 / 68872
leukemia	0		0 / 94479
liver tumor	0		0 / 96023
lung tumor	0		0 / 102765
lymphoma	0		0 / 72196
non-neoplasia	0		0 / 96623
normal	0		0 / 3328811
ovarian tumor	0		0 / 76185
pancreatic tumor	0		0 / 105004
primitive neuroectodermal tumor...	0		0 / 127001
prostate cancer	0		0 / 103844
retinoblastoma	0		0 / 46439
skin tumor	0		0 / 125373
soft tissue/muscle tissue tumor	0		0 / 125265
uterine tumor	11		1 / 90107

Breakdown by Developmental Stage

Pool name/ tissue	Transcripts per million	Spot intensity	Gene EST/Total EST in pool
			Hs.447993
embryoid body	0		0 / 69969
blastocyst	0		0 / 61448
fetus	0		0 / 556978
neonate	0		0 / 31070
infant	0		0 / 23511
juvenile	0		0 / 55574
adult	0		1 / 1921829

Supplementary Figure S9: ASTL expressed sequence tags provided in the Unigene database. EST profiles are barometers of gene expression patterns as inferred from EST counts and tissue sources of the cDNA libraries probed. The only ASTL hit seen in the Unigene database is in a uterine tumor identified while performing a shotgun sequencing of the human transcriptome using a mini uterine tumor library cloned in a pUC 18 vector. The sequence was submitted by the Ludwig Institute for Cancer Research in Brazil. Note that no signal is seen in the ovary. This is likely due to the relative rarity of ASTL messages in the ovary due to the stage specific expression of SAS1B in secondary follicle oocytes and beyond, the pool of which is few in number in normally menstruating woman as well as the age and the cyclicity of the deposits.
